# Runx/Cbfβ complexes protect group 2 innate lymphoid cells from exhausted-like hyporesponsiveness during allergic airway inflammation

**DOI:** 10.1038/s41467-019-08365-0

**Published:** 2019-01-25

**Authors:** Chizuko Miyamoto, Satoshi Kojo, Motoi Yamashita, Kazuyo Moro, Georges Lacaud, Katsuyuki Shiroguchi, Ichiro Taniuchi, Takashi Ebihara

**Affiliations:** 1 Laboratory for Transcriptional Regulation, RIKEN Center for Integrative Medical Sciences (IMS), 1-7-22 Suehiro-cho, Tsurumi-ku, Yokohama, 230-0045 Japan; 2 Laboratory for Innate Immune Systems, RIKEN Center for Integrative Medical Sciences (IMS), 1-7-22 Suehiro-cho, Tsurumi-ku, Yokohama 230-0045 Japan; 3 Stem Cell Biology Group, Cancer Research UK Manchester Institute, The University of Manchester, Wilmslow Road, Manchester, M20 4BX UK; 4 Laboratory for Immunogenetics, RIKEN Center for Integrative Medical Sciences (IMS), 1-7-22 Suehiro-cho, Tsurumi-ku, Yokohama 230-0045 Japan; 5 Laboratory for Prediction of Cell Systems Dynamics, RIKEN Center for Biosystems Dynamics Research (BDR), Suita, Osaka, 565-0874 Japan; 6 JST PRESTO, Kawaguchi, 332-0012 Japan

## Abstract

Group 2 innate lymphoid cells (ILC2s) have tissue-resident competence and contribute to the pathogenesis of allergic diseases. However, the mechanisms regulating prolonged ILC2-mediated T_H_2 cytokine production under chronic inflammatory conditions are unclear. Here we show that, at homeostasis, Runx deficiency induces excessive ILC2 activation due to overly active GATA-3 functions. By contrast, during allergic inflammation, the absence of Runx impairs the ability of ILC2s to proliferate and produce effector T_H_2 cytokines and chemokines. Instead, functional deletion of Runx induces the expression of exhaustion markers, such as IL-10 and TIGIT, on ILC2s. Finally, these ‘exhausted-like’ ILC2s are unable to induce type 2 immune responses to repeated allergen exposures. Thus, Runx confers competence for sustained ILC2 activity at the mucosa, and contributes to allergic pathogenesis.

## Abstract (web summary)

Group 2 innate lymphoid cells (ILC2) are important mediators for allergy, but how ILC2 are regulated under chronic inflammation is still unclear. Here the authors show that Runx transcription factors, which normally suppresses ILC2 activation at steady state, help promote ILC2 activation and type 2 cytokine production in lung allergy mouse models.

## Introduction

Innate lymphoid cells (ILCs) are enriched in mucosal tissues, where they function as sentinel cells at the front line of host defense^1^. Although ILCs do not possess rearranged antigen-specific receptors, they exert a helper function similar to T_H_ cells by producing helper cytokines. ILCs are categorized into three main subsets: T_H_1-like ILC1s, T_H_2-like ILC2s, and T_H_17/T_H_22-like ILC3s^2–6^. Recently, another subset of ILCs named regulatory ILCs (ILCregs) has been reported to provide an immune suppressive function by producing IL-10 in the intestine^7^.

ILC2s are the main population producing IL-5, which recruits eosinophils into tissues under healthy conditions^8^. Upon allergic stimulation, ILC2s are activated by IL-25, IL-33, and TSLP from damaged epithelial cells, IL-2, IL-4, and IL-9 from other haematopoietic cells or from ILC2s themselves, neuropeptides, and lipid mediators^1,9–11^. Activated ILC2s contribute to deterioration of allergic diseases by producing high levels of IL-5 and IL-13, both of which enhance the T_H_2 induction and inflammation mediated by eosinophils. An ILC2 subset producing IL-10 (ILC2_10_s) in regions of chronic or severe allergic inflammation is associated with reduction of eosinophils in the lung by unknown mechanisms^12^.

Recurrent stimulation influences the biological properties of ILC2s, as well as T cells. After the effector phase, T cells can become long-lived memory T cells in the tissues or lymph nodes, where they are reactivated by the same antigen. A similar recall response was also observed in ILC2s pre-activated with IL-33 or allergens^13^. In contrast, T cells at sites of chronic inflammation become exhausted and lose their effector functions, including cytokine production and proliferation, in response to repeated stimulation^14^. PD-1, which is a T cell exhaustion marker, is induced on activated ILC2s and negatively regulates this cell pool^15^. However, PD-1^+^ ILC2s are not considered exhausted because they continue to produce IL-5 normally. Thus, ILC2s with a hyporesponsive phenotype similar to exhausted T cells have not yet been identified.

The mammalian Runx transcription factor protein family is composed of Runx1, Runx2, and Runx3. Each Runx protein requires heterodimer formation with Cbfβ to bind DNA^16^. Runx3 is the main family member expressed in all ILC subsets and is indispensable for the differentiation and function of the ILC1 and ILC3 subsets^17^. However, depletion of Runx3 alone has little effect on ILC2 differentiation, probably due to the redundant functions of other Runx proteins, such as Runx1, which is expressed in ILC2s. Thus, the function of Runx/Cbfβ complexes in ILC2s has not been clarified.

Here, we show that Runx/Cbfβ complexes are not necessary for ILC2 differentiation but modulate ILC2 function. At steady state, Runx-deficient ILC2s are activated and aberrantly secrete IL-5, resulting in increased eosinophil recruitment to the lung. However, after allergic stimulation, ILC2s lacking Runx fail to proliferate and produce various cytokines and chemokines but have increased expression of IL-10 and TIGIT, which are known markers of exhausted T cells. We explore the existence of IL-10^+^ TIGIT^+^ ILC2s with low reactivity in the physiological setting and find that severe subacute allergic inflammation induces the emergence of hyporesponsive IL-10^+^ TIGIT^+^ ILC2s, and that this effect is enhanced by Cbfβ deficiency. Collectively, our data reveal that Runx/Cbfβ complexes are required to prevent ILC2s from entering an exhausted-like functional state under allergic conditions.

## Results

### Runx is not required for development of ILC2s

**Fig. 1:**
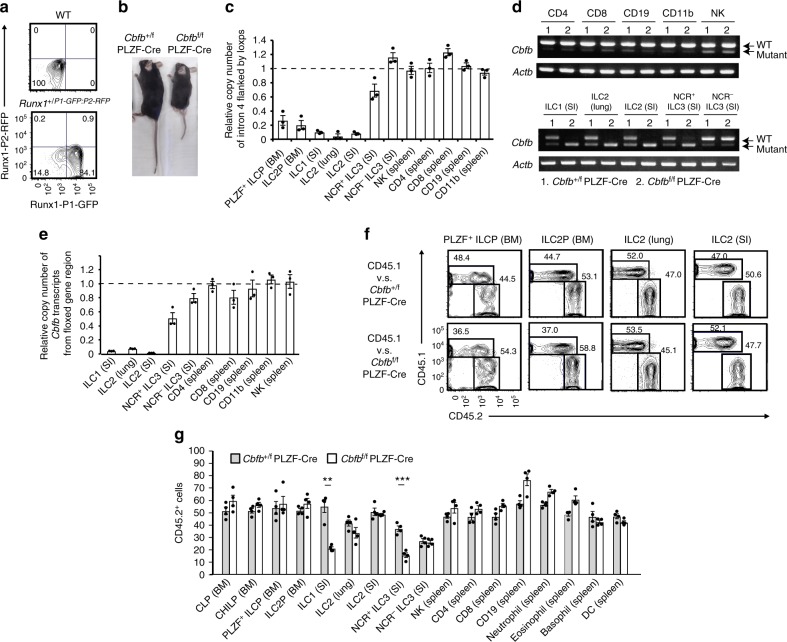
Cbfβ is not necessary for ILC2 differentiation. **a** Flow cytometry analysis of Runx1-GFP expression from the P1 promotor and Runx1-RFP from the P2 promotor by ILC2s (CD45^+^ CD3^–^ CD19^–^ CD127^+^ CD25^+^ ST2^+^) in the lungs of WT (top) and Runx1^+/P1-GFP: P2-RFP^ mice (bottom). **b** Gross appearance of the *Cbfb*^+/f^ PLZF-Cre (left) and *Cbfb*^f/f^ PLZF-Cre mice (right). **c** Quantitative PCR analysis of the relative copy numbers of the floxed genes (loxP site, loxps) located in *Cbfb* intron 4 compared to those of the unfloxed genes in *Cbfb* intron 4 in the indicated cell populations of *Cbfb*^f/f^ PLZF-Cre mice. **d** RT-PCR assay of *Cbfb* transcripts with or without a mutation in the indicated cell populations of the *Cbfb*^+/f^ PLZF-Cre (1) and *Cbfb*^f/f^ PLZF-Cre mice (2). **e** Quantitative PCR analysis of the relative copy numbers of *Cbfb* transcripts from the floxed *Cbfb* gene locus in the indicated cell populations of *Cbfb*^f/f^ PLZF-Cre mice. **f**, **g** Chimerism of common lymphoid progenitor (CLP: CD45^+^ Lin^–^ cKit^lo^ CD127^+^ sca1^lo^ Flt3^+^ α4β7^–^), CHILP (CD45^+^ Lin^–^ CD127^+^ α4β7^+^ Flt3^–^ CD25^–^), PLZF^+^ ILCP (CD45^+^ Lin^–^ cKit^+^ CD127^+^ α4β7^+^ PLZF-GFP^+^), ILC2 precursor (ILC2P: CD45^+^ Lin^–^ CD127^+^ α4β7^+^ Flt3^–^ CD25^+^) cells, ILC1s (CD45^+^ CD3^–^ CD19^–^ NK1.1^+^ NKp46^+^ CD127^+^), ILC2s (CD45^+^ CD3^–^ CD19^–^ CD127^+^ GATA-3^+^), and NCR^+ or –^ (NKp46^+or –^) ILC3s (CD45^+^ CD3^–^ CD19^–^ CD127^+^ RORγt^+^), NK cells, CD4^+^ T cells, CD8^+^ T cells, CD19^+^ B cells, neutrophils, eosinophils, basophils, and DCs (CD11b^+^ CD11c^+^) from the indicated tissues of lethally irradiated host mice (CD45.1^+^) reconstituted with a mixture (1:1) of bone marrow cells from the wild type (CD45.1^+^) and *Cbfb*^+/f^ PLZF-Cre or *Cbfb*^f/f^ PLZF-Cre (CD45.2^+^) mice. BM bone marrow, SI small intestine lamina propria lymphocytes. Numbers adjacent to the outlined areas in **c** indicate the percentages of CD45.1^–^/CD45.2^+^ (mutant donor) or CD45.1^+^ CD45.2^–^ (wild type) cells. In **g**, **p < 0.01 and ***p < 0.001 by Student’s *t*-test. Data are representative of at least two independent experiments (mean ± s.d. of three technical replicates in **c**, **e**, mean ± s.d. of 4 mice in **f**, **g**)

Of all of the ILCs and ILC progenitors, the highest *Runx1* and *Runx3* mRNA expression levels are found in the common precursor to ILCs (ILCPs), which is marked by stage-specific PLZF expression and can differentiate into ILC1s, ILC2s, and NCR^+^ ILC3s (a subpopulation of ILC3s)^17^. Analysis of Runx3 reporter mice suggests that downregulation of Runx3 may be required for PLZF^+^ ILCPs to enter the ILC2 pathway, whereas ILC1s and ILC3s require intermediate to high levels of Runx3 for their differentiation^17^. To precisely examine Runx1 protein expression in ILC subsets and progenitors, we took advantage of Runx1^+/P1-GFP: P2-RFP^ mice, in which GFP or RFP was driven from the distal (P1) or proximal (P2) *Runx1* promoter, respectively^18^. PLZF^+^ ILCPs utilized both the P1 and P2 promoters for high Runx1 expression, although ILC2s in the lung and intestine expressed Runx1 from the P1 promoter to a greater extent than ILC1s and ILC3s in the intestine (Fig. 1a and Supplementary Fig. 1). Thus, Runx1 is expressed by ILC2s despite low Runx3 expression.

To assess the roles of Runx/Cbfβ complexes in the function and differentiation of PLZF^+^ ILCPs into ILC2s, we deleted Cbfβ in the PLZF^+^ ILCPs using PLZF-Cre (*Cbfb*^f/f^ PLZF-Cre mice). This strategy should result in a complete loss of any Runx protein function in the descendant ILC subsets, including ILC1s, ILC2s, and NCR^+^ ILC3s. The *Cbfb*^f/f^ PLZF-Cre mice were born and grew to adults, although they were smaller than their littermate controls (Fig. 1b) and had bone distortion and difficulty walking. The defects in bone formation may be explained by the loss of Runx2, which is critical for osteoblast differentiation, because PLZF is expressed in osteoblasts prior to Runx2 induction^19^. Then, we examined which lymphocytes suffered from the Cbfβ mutation in the *Cbfb*^f/f^ PLZF-Cre mice to validate our system. We observed highly efficient deletion of *Cbfb* in the PLZF^+^ ILCP and ILC2 progenitors (ILC2Ps) from the bone marrow, ILC2s from the lung and small intestine, and ILC1s from the small intestine and partial *Cbfb* deletion in NCR^+^ ILC3s from the small intestine in the *Cbfb*^f/f^ PLZF-Cre mice (Fig. 1c). However, the *Cbfb* genes in the CD4^+^ T, CD8^+^ T, NK, and B cells were not greatly affected in the *Cbfb*^f/f^ PLZF-Cre mice. Wild type *Cbfb* transcripts were efficiently deleted in the ILC subsets of the *Cbfb*^f/f^ PLZF-Cre mice, although subtle transcript expression of mutated *Cbfb* was detected in the non-ILC populations (Fig. 1d, e). Thus, the Cbfβ dysfunction is specifically induced in ILC subsets among haematopoietic cell populations of the *Cbfb*^f/f^ PLZF-Cre mice.

The specificity of this deletion effect for ILC subsets is surprising, because haematopoietic stem cells are fate-mapped by PLZF expression^20^. To confirm previous data, we crossed *Cbfb*^f/f^ PLZF-Cre mice with Rosa26-tdTomato mice in which PLZF-Cre expression in the progenitor cells could be followed by tdTomato expression in the *Cbfb*^f/f^ PLZF-Cre mice. As previously described, most haematopoietic cells were labeled with tdTomato in the mice (Supplementary Fig. 2). However, the *Cbfb* gene locus flanked by loxps was quite intact in the tdTomato^+^ cells of the major haematopoietic cell populations. These data indicate that PLZF-Cre can reach and excise the Rosa26 locus but not the *Cbfb* locus in the progenitors of the major haematopoietic populations, probably due to the tight chromatin structure of the *Cbfb* locus.

Next, we assessed the cell-intrinsic effect of Cbfβ deletion on the differentiation of PLZF^+^ ILCPs, ILC2Ps, and ILC2s in the lung and intestine by performing competitive bone marrow reconstitution experiments. Fifty percent CD45.2^+^ *Cbfb*^+/f^ PLZF-Cre or *Cbfb*^f/f^ PLZF-Cre bone marrow cells were adoptively transferred together with fifty percent CD45.1^+^ competitors into lethally irradiated CD45.1^+^ mice. The PLZF^+^ ILCPs, ILC2P, and ILC2s developed normally in the absence of Cbfβ function (Fig. 1f), although *Cbfb*^f/f^ PLZF-Cre bone marrow cells differentiated into fewer ILC1s and NCR^+^ ILC3s in the small intestine lamina propria lymphocytes (LPL) than *Cbfb*^+/f^ PLZF-Cre bone marrow cells (Fig. 1g). Differentiation of haematopoietic cells other than ILCs was not abrogated in the *Cbfb*^f/f^ PLZF-Cre mice (Fig. 1g). Thus, Cbfβ is dispensable for differentiation of PLZF^+^ ILCPs and ILC2s in peripheral tissues.

### Runx restrains steady-state ILC2 activation

**Fig. 2:**
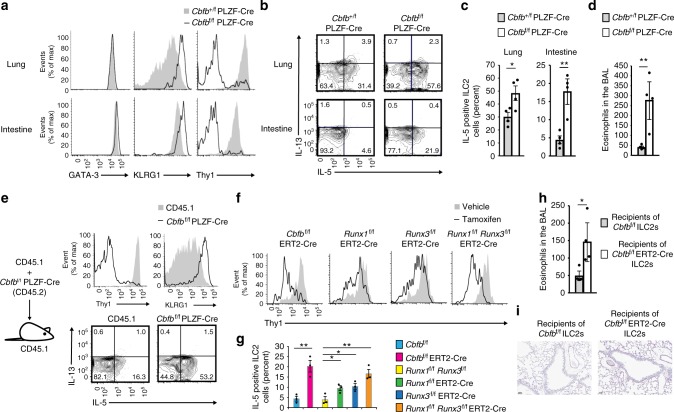
Runx/Cbfβ complexes suppress ILC2 activity in the steady state of the lung and intestine. **a** Flow cytometry analysis of GATA-3, KLRG1, and Thy1 expression by ILC2s (CD45^+^ CD3^–^ CD19^–^ CD127^+^ CD25^+^ ST2^+^ KLRG1^+^) from the lung or small intestine of the *Cbfb*^+/f^ PLZF-Cre or *Cbfb*^f/f^ PLZF-Cre mice. **b** Flow cytometry analysis of IL-5 and IL-13 expression by ILC2s from the lung or small intestine of the *Cbfb*^+/f^ PLZF-Cre or *Cbfb*^f/f^ PLZF-Cre mice. **c** The frequency of IL-5-producing cells in ILC2s from the lung or small intestine of the *Cbfb*^+/f^ PLZF-Cre or *Cbfb*^f/f^ PLZF-Cre mice was determined by flow cytometry as in **b**. **d** Absolute number of eosinophils in the bronchoalveolar lavage (BAL) fluid from the *Cbfb*^+/f^ PLZF-Cre or *Cbfb*^f/f^ PLZF-Cre mice. **e** Flow cytometry analysis of the expression of the indicated proteins by CD45.1^+^ or CD45.2^+^ *Cbfb*^f/f^ PLZF-Cre ILC2s from lethally irradiated host mice (CD45.1^+^) reconstituted with a mixture (1:1) of bone marrow cells from the wild type (CD45.1^+^) and *Cbfb*^f/f^ PLZF-Cre (CD45.2^+^) mice. **f** Flow cytometry analysis of Thy1 expression by ILC2s from the small intestine of the *Cbfb*^f/f^ ERT2-Cre, *Runx1*^f/f^ ERT2-Cre, Runx3^f/f^ ERT2-Cre, and Runx1^f/f^ Runx3^f/f^ ERT2-Cre mice treated with the vehicle control or tamoxifen. **g** The frequency of IL-5-producing cells in the ILC2s from the small intestine of the indicated mice treated with tamoxifen as in **f** was determined by flow cytometry as in **c**. **h**, **i** *Rag2*^–/–^ *Il2rg*^–/–^ mice were transferred with 1 × 10^6^ *Cbfb*^f/f^ or *Cbfb*^f/f^ ERT2-Cre ILC2s expanded with IL-2 and IL-7 in vitro and then treated with tamoxifen. Absolute numbers of eosinophils in the BAL fluid from the indicated recipients (**h**). Histology of PAS-positive epithelial cells in the indicated recipients (**i**). Numbers in the quadrants indicate the percentages of cells in each quadrant (**b**, **e**). In **c**, **d**, **g**, **h**, *p *<* 0.05 and **p < 0.01 by Student’s *t*-test. Data are representative of at least two independent experiments (mean ± s.d. of four mice in **c**, **d**, mean ± s.d. of three mice in **g**, **h**)

We investigated whether Cbfβ deficiency affected basal ILC2 activity in the steady state of the lung. To this end, first we examined KLRG1 activation marker expression on the ILC2s in the lung and intestine of the *Cbfb*^+/f^ PLZF-Cre and *Cbfb*^f/f^ PLZF-Cre mice^21^. ILC2s from the *Cbfb*^f/f^ PLZF-Cre mice expressed more KLRG1 than those from the *Cbfb*^+/f^ PLZF-Cre mice (Fig. 2a). Further phenotypic analysis demonstrated that downregulation of Thy1 occurred on the *Cbfb*^f/f^ PLZF-Cre ILC2s like ILC2s stimulated with IL-25^22^. In addition, the *Cbfb*^f/f^ PLZF-Cre ILC2s produced elevated levels of IL-5, which were correlated with enhanced recruitment of eosinophils to the bronchoalveolar space (Fig. 2b–d). IL-25 stimulation induces inflammatory ILC2s defined as KLRG1^Hi^ Thy1^Lo^ ST2 (IL-33Ra)^–^ ILC2s^22^. However, the *Cbfb*^f/f^ PLZF-Cre ILC2s were different from these inflammatory ILC2s, because expression of cytokine receptors, including ST2, was not significantly altered by Cbfβ deficiency (Supplementary Fig. 3). To investigate whether ILC2 activation in the *Cbfb*^f/f^ PLZF-Cre mice is cell intrinsic or extrinsic, we performed bone marrow competition assays with CD45.1^+^ competitor cells as described above. *Cbfb*^f/f^ PLZF-Cre ILC2s in the recipient lungs showed increased KLRG1 expression, decreased Thy1 expression, and IL-5 overproduction compared to those of the competitor cells (Fig. 2e). These data indicate that Cbfβ suppresses the basal activity of ILC2s in a cell intrinsic manner.

To address the question of which Runx proteins contribute to the ILC2 activation phenotype in the absence of Cbfβ, we sought to generate *Runx1*^f/f^ PLZF-Cre and *Runx3*^f/f^ PLZF-Cre mice. However, most of the *Runx1*^f/f^ PLZF-Cre mice died soon after birth for unknown reasons. Therefore, instead we generated *Cbfb*^f/f^ ERT2-Cre, *Runx1*^f/f^ ERT2-Cre, *Runx3*^f/f^ ERT2-Cre, and *Runx1*^f/f^ *Runx3*^f/f^ ERT2-Cre mice. Inducible deletion of Cbfβ or both Runx1 and Runx3 by oral tamoxifen administration led to a robust reduction of Thy1 expression and an increase of IL-5 production in the intestine (Fig. 2f, g). On the other hand, single deletion of either Runx1 or Runx3 had little effect on Thy1 expression and IL-5 production by the ILC2s (Fig. 2f, g). These results suggest that Runx1 and Runx3 must work jointly and together with Cbfβ to repress ILC2 functions under steady-state conditions.

To investigate the physiological impact of enhanced ILC2 activity resulting from Cbfβ deficiency on the steady state of the lung, we adoptively transferred *Cbfb*^f/f^ ILC2s or *Cbfb*^f/f^ ERT2-Cre ILC2s into *Rag2*^–/–^ *Il2rg*^–/–^ mice, which were treated with tamoxifen after transfer. At 3–4 weeks after the tamoxifen treatment, almost no damaged PAS^+^ epithelial cells were observed in the lungs of the *Cbfb*^f/f^ ERT2-Cre ILC2 recipient mice, although the *Cbfb*^f/f^ ERT2-Cre ILC2s increased the eosinophil numbers in the bronchoalveolar lavage (BAL) fluid (Fig. 2h, i). Thus, Cbfβ-deficient ILC2s are not sufficiently active to acutely damage lung epithelial cells but instead contribute to subsymptomatic eosinophil infiltration into the bronchoalveolar space.

### Runx antagonizes GATA-3 function in steady-state ILC2s

**Fig. 3:**
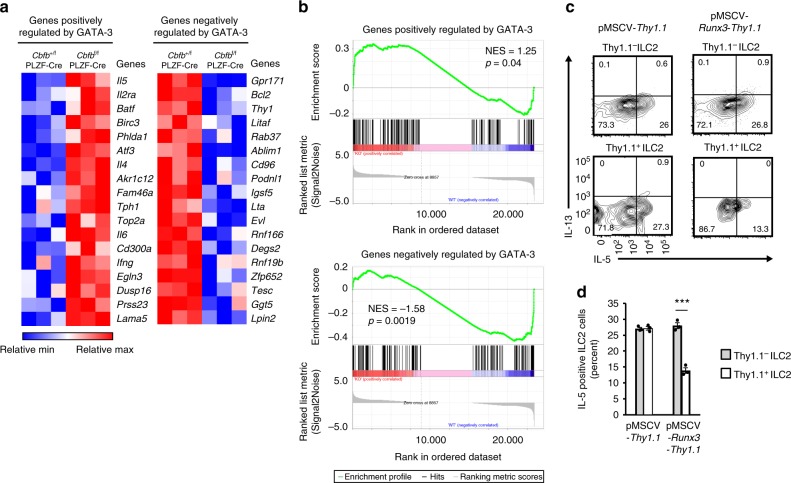
Runx proteins inhibit the gene expression regulated by GATA-3 in ILC2s at steady state. **a** Heat map of the expression of selected genes positively (left) or negatively (right) regulated by GATA-3 in ILC2s as determined by RNA sequence analysis. **b** Gene set enrichment analysis of genes positively (top) or negatively regulated by GATA-3 performed on a gene set that was differentially expressed in *Cbfb*^f/f^ PLZF-Cre ILC2s compared to *Cbfb*^+/f^ PLZF-Cre ILC2s. **c**, **d** Host mice (CD45.1^+^) were lethally irradiated and reconstituted with CD45.2^+^ bone marrow cells that were retrovirally transduced with the control vector, pMSCV-*Thy1.1* (left) or pMSCV-*Runx3*-*Thy1.1* (right). Flow cytometry analysis of IL-5 and IL-13 expression by untransduced Thy1.1^–^ (top) or transduced Thy1.1^+^ (bottom) donor CD45.2^+^ ILC2s (CD45^+^ CD3^–^ CD19^–^ CD127^+^ CD25^+^ KLRG1^+^) from the lungs of the host mice (**c**). The frequency of IL-5-producing cells in the indicated ILC2s was determined by flow cytometry as in **c** (**d**). Numbers in quadrants of the bottom plots indicate the percentages of cells in each quadrant. In **d**, ***p < 0.001 by Student’s *t*-test. Data are representative of at least two independent experiments (mean ± s.d. of three mice in **d**)

A previous study demonstrated that Runx proteins antagonized GATA-3 function in T cells by directly binding to GATA-3^23^. We hypothesized that the same inhibitory mechanism by Runx proteins might function in ILC2s to suppress IL-5 production by antagonizing GATA-3 activity. To test this hypothesis, we performed RNA sequence analysis of ILC2s from the lungs of *Cbfb*^+/f^ PLZF-Cre and *Cbfb*^f/f^ PLZF-Cre mice and analyzed the expression profiles of genes that were positively or negatively regulated by GATA-3 in ILC2s^24^. If our hypothesis was correct, the function of GATA-3 as a transcription factor would be enhanced in the absence of Cbfβ. Deletion of Cbfβ in ILC2s led to upregulation of 18 of 156 genes (11.5%) that were positively regulated by GATA-3 and downregulation of 30 of 151 genes (19.8%) that were negatively regulated by GATA-3 (Fig. 3a, Supplementary Data 1). GATA-3 positively regulated IL-5 expression, and this effect was augmented by Cbfβ deficiency. In contrast, GATA3 was a negative regulator of Thy1 expression, which was even more inhibited in the absence of Cbfβ. In addition, a set of genes positively or negatively regulated by GATA-3 was significantly enriched in the Cbfβ-deficient ILC2s (Fig. 3b). Given that Runx/Cbfβ complexes antagonize GATA-3 function, over-expression of Runx protein should suppress IL-5 production by ILC2s. To explore this possibility, CD45.2^+^ C57BL/6 bone marrow cells were transduced with a retroviral vector encoding Runx3, followed by IRES-Thy1.1 and were adoptively transferred into lethally irradiated CD45.1^+^ congenic mice. At 8 weeks after transfer, the Thy1.1^+^ ILC2s over-expressing Runx3 produced less IL-5 than the Thy1.1^–^ non-transduced cells or the Thy1.1^+^ cells transduced with the control vector (Fig. 3c, d). With the same over-expression system, we confirmed that GATA-3 over-expression in ILC2s increased IL-5 production, which was inhibited by Runx3 (Supplementary Fig. 4). Collectively, the Runx/Cbfβ complexes suppress the constitutive activity of ILC2s at least in part by inhibiting the function of GATA-3.

### Runx protects ILC2s from exhausted-like hyporesponsiveness

**Fig. 4:**
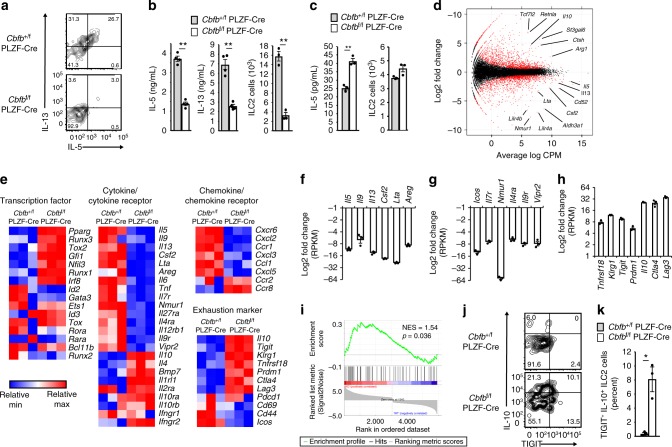
Cbfβ-deficient ILC2s show an exhausted-like phenotype after cytokine stimulation. ILC2s (CD45^+^ CD3^–^ CD19^–^ CD127^+^ CD25^+^ ST2^+^ KLRG1^+^) were sorted from the lungs of *Cbfb*^+/f^ PLZF-Cre or *Cbfb*^f/f^ PLZF-Cre mice and cultured with IL-2 and IL-33 (**a**, **b**, **d**–**k**) or IL-2 and IL-7 (**c**). The number of days is indicated below. **a** Flow cytometry analysis of IL-5 and IL-13 expression by ILC2s from the indicated mice cultured for 3 days. **b**, **c** The IL-5 (**b**, left; **c**, left) and IL-13 (**b**, middle) concentrations in the culture supernatants from the indicated mouse ILC2s on day 4 and the absolute number of cultured ILC2s (**b**, right; **c**, right) from the indicated mice on day 6. **d** MA plots of genes expressed in *Cbfb*^f/f^ PLZF-Cre ILC2s versus *Cbfb*^+/f^ PLZF-Cre ILC2s cultured with IL-5 and IL-13 for 4 days. Red dots indicate genes that were significantly differentially expressed (false discovery rate < 0.05). **e** Heat map of selected genes expressed in the cultured *Cbfb*^f/f^ PLZF-Cre ILC2s versus *Cbfb*^+/f^ PLZF-Cre ILC2s (**e**). **f**, **g**, **h** Mean fold change of RPKM values of the indicated genes in *Cbfb*^f/f^ PLZF-Cre ILC2s versus *Cbfb*^+/f^ PLZF-Cre ILC2s. **i** Gene set enrichment analysis of signature genes observed in exhausted CD8^+^ T cells performed on a gene set that was differentially expressed in *Cbfb*^f/f^ PLZF-Cre ILC2s stimulated with IL-33 in vitro compared to *Cbfb*^+/f^ PLZF-Cre ILC2s. **j** Flow cytometry analysis of TIGIT and IL-10 expression by cultured ILC2s from the indicated mice on day 6. **k** The frequency of TIGIT^+^ IL-10^+^ cells in the indicated ILC2s was determined by flow cytometry as in **j**. Numbers indicate the percentages of cells in each quadrant (**a**, **j**). In **b**, **c**, **k**, *p *<* 0.05 and **p < 0.01 by Student’s *t*-test. Data are representative of at least two independent experiments (mean ± s.d. of four mice in **b**, **c** and of three mice in **k**)

To determine how Cbfβ regulates ILC2 effector functions after activation, first we cultured ILC2s from the lungs of *Cbfb*^+/f^ PLZF-Cre and *Cbfb*^f/f^ PLZF-Cre mice with IL-2 and IL-33, which is a cytokine combination that is the most potent ILC2 stimulator and is critical for establishment of allergic inflammation. We expected that this cytokine stimulation would lead to unleashed production of T_H_2 cytokines by activated ILC2s due to Cbfβ deficiency. Surprisingly, ILC2s lacking Cbfβ secreted decreased IL-5 and IL-13 levels and did not grow well (Fig. 4a, b) in response to IL-2 and IL-33 in vitro. This hyporesponsiveness of ILC2s did not occur when the basal ILC2 activity was maintained by IL-2 and IL-7 without IL-33, which is a strong inducer of allergy (Fig. 4c). To understand the mechanism of the low reactivity of Cbfβ-deficient ILC2s to IL-33, we conducted RNA sequencing analysis of in vitro-activated lung ILC2s from *Cbfb*^+/f^ PLZF-Cre and *Cbfb*^f/f^ PLZF-Cre mice (Fig. 4d–i and Supplementary Data 2). The gene expression profiles of the transcription factors expressed in the ILC subsets were essentially comparable between the *Cbfb*^+/f^ PLZF-Cre and *Cbfb*^f/f^ PLZF-Cre ILC2s with some exceptions, such as *Gfi1*, *Nfil3*, and *Irf8* (Fig. 4e). However ablation of Cbfβ in the ILC2s led to reduced expression of many effector cytokines and chemokines and their receptors. Surprisingly, crucial ILC2 cytokines, including *Il5*, *Il9*, *Il13*, *Csf2* encoding GM-CSF, *Lta*, and *Areg* encoding amphiregulin, were all downregulated in ILC2s lacking Cbfβ (Fig. 4e, f). To remain activated, ILC2s require activating signals through cell surface receptors, including *IL7r*, *Il9r, IL4ra, Icos*, and *Nmur1*, which all show reduced expression in the absence of Cbfβ (Fig. 4e, g). In contrast, Cbfβ-deficient ILC2s expressed high levels of T cell exhaustion markers, including the *Tnfrsf18* encoding GITR, *Klrg1*, *Tigit*, *Prdm1* encoding Blimp1, *Il10*, *Ctla4*, and *Lag3* genes (Fig. 4e, h)^14^. Gene set enrichment analysis indicated that Cbfβ-deficient ILC2s stimulated with IL-33 had a signature of exhausted CD8^+^ T cells (Fig. 4i). Since the TIGIT and IL-10 expression levels were low in the control ILC2s, we thought that upregulation of TIGIT and IL-10 might be good marker for this hyporesponsiveness and confirmed the elevated expression of the IL-10 and TIGIT proteins in activated-ILC2s lacking Cbfβ in vitro (Fig. 4j, k). Collectively, ILC2s present in the lungs of the *Cbfb*^f/f^ PLZF-Cre mice show hyporesponsiveness to cytokine stimulation in vitro and acquire unique gene expression signatures similar to those observed in exhausted T cells.

IL-10 and inhibitory signals through TIGIT could be responsible for the low reactivity of the *Cbfb*^f/f^ PLZF-Cre ILC2s against IL-33 stimulation. However, the IL-10 concentration in the culture supernatant of *Cbfb*^f/f^ PLZF-Cre ILC2s was not high enough to detect the slight inhibitory effect on ILC2s (Supplementary Fig. 5a, b). Neutralization of IL-10 did not cancel the low reactivity of the *Cbfb*^f/f^ PLZF-Cre ILC2s against IL-33 (Supplementary Fig. 5c). In addition, our RNA sequence analysis showed that the expression levels of TIGIT ligands, such as CD112 and CD155, were quite low in ILC2s; the RPKM values of these molecules were less than 1. Therefore, the low reactivity of ILC2s without Cbfβ function did not result from increased IL-10 or TIGIT expression.

To determine what Runx proteins were responsible for this hyporesponsiveness to cytokine stimulation, we deleted Cbfβ, Runx1, Runx3, or both Runx1 and Runx3 by oral tamoxifen administration to *Cbfb*^f/f^ ERT2-Cre, *Runx1*^f/f^ ERT2-Cre, *Runx3*^f/f^ ERT2-Cre, or *Runx1*^f/f^ *Runx3*^f/f^ ERT2-Cre mice as described above and stimulated the intestinal ILC2s with IL-2 and IL-33 in vitro. Runx1 or Runx3 single deletion resulted in minor changes in IL-5 and IL-13 production by the ILC2s (Supplementary Fig. 6a, b). However, when Runx1 and Runx3 were both deleted, IL-5 and IL-13 production were both reduced to the level observed in the Cbfβ-deleted ILC2s. In addition, we performed ChIP sequence analysis for Runx1 and Runx3 binding in ILC2s activated with IL-33 to examine the differential functions of Runx1 and Runx3 in the hyporesponsiveness of ILC2s. However, generally the binding patterns were comparable between Runx1 and Runx3 (Supplementary Fig. 6c, d). Collectively, both Runx1 and Runx3 serve as inhibitors of the exhausted-like phenomenon in cooperation with Cbfβ.

### GATA-3-dependent and GATA-3-independent functions of Runx in ILC2s

**Fig. 5:**
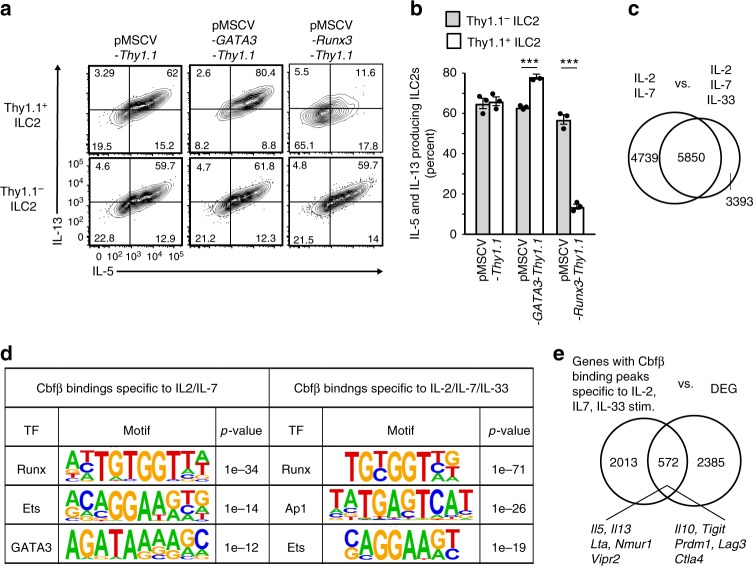
Differential functions of the GATA-3 and Runx proteins in activated ILC2s. **a** Flow cytometry analysis of IL-5 and IL-13 production by ILC2s transfected with the indicated retroviral vectors and cultured with IL-2, IL-7, and IL-33. Thy1.1 is a surrogate marker for transduction. **b** The frequency of cells producing both IL-5 and IL-13 in the indicated ILC2s was determined by flow cytometry as in **a**. **c** Venn diagram showing the number of binding events in ILC2s cultured with IL-2/IL-7 or IL-2/IL-7/IL-33 in vitro. **d** Motif analysis of Cbfβ binding peaks specific to the indicated ILC2s. **e** Venn diagram showing the number of genes with Cbfβ binding peaks specific to ILC2s cultured with IL2/IL-7/IL33 in vitro and differentially expressed genes (DEG) in *Cbfb*^f/f^ PLZF-Cre ILC2s compared to *Cbfb*^+/f^ PLZF-Cre ILC2s. The numbers indicate the percentages of cells in each quadrant (**a**). In **b**, ***p < 0.001 by Student’s *t*-test. Data are representative of at least two independent experiments (mean ± s.d. of three mice in **b**)

We sought to investigate how pre-activated *Cbfb*^f/f^ PLZF-Cre ILC2s at steady state showed hyporesponsiveness to IL-33 stimulation. First, we hypothesized that GATA-3 overactivation due to the absence of antagonizing effects by the Runx protein pushed ILC2s into an overactivated hypofunctional state. However, over-expressed GATA-3 still enhanced IL-5 and IL-13 production by ILC2s in response to IL-33 in vitro (Fig. 5a, b). To examine whether the Runx proteins inhibited ILC2 activity by antagonizing GATA-3 as observed in the steady-state ILC2s, we evaluated cytokine production by IL-33-stimulated ILC2s over-expressing Runx3. Interestingly, Runx3 over-expression dampened cytokine production by ILC2s in response to IL-33. Thus, the Runx proteins apparently inhibit ILC2 activity in a dose-dependent manner even after IL-33 stimulation.

**Fig. 6:**
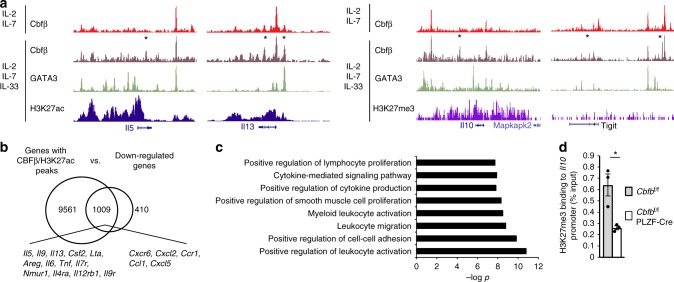
Enhancer and repressor activities of Runx proteins in ILC2s stimulated with IL-33. **a** Tracks of Cbfβ ChIP sequence traces in ILC2s cultured with IL-2 and IL-7 (top) and Cbfβ, GATA-3, H3K27 acetylation (ac), and H3K27 trimethylation (me3) ChIP sequence traces in ILC2s cultures with IL-2, IL-7, and IL-33 (bottom three). Asterisks(*) indicates a differential peak in IL2s cultured with IL-2, IL-7, and IL-33 compared to ILC2s cultured with IL-2 and IL-7. **b** Venn diagram showing the number of genes with Cbfβ binding peaks marked by H3K27ac peaks (left) and the downregulated genes in the Cbfβ-deficient ILC2s as determined by RNA sequence analysis (right). **c** Gene ontology enrichment analysis using the top 200 downregulated genes out of the 1009 genes in **a** (middle). Bar plots indicates the –log *p* value for GO term enrichment. **d** H3K27me3 binding to the *Il10* promoter locus in the indicated mouse ILC2s cultured with IL-2. IL-7, and IL-33. In **d**, *p < 0.05 by Student’s *t*-test. Data are representative of two independent experiments (mean ± s.d. of three mice in **d**)

Since high transcription factor expression is not always required for enhancer or repressor function, we hypothesize that a dose-independent and GATA-3-independent function of Runx proteins should exist as an epigenetic modulator for ILC2s to normally respond to IL-33. To test this possibility, we examined Cbfβ binding in ILC2s cultured with or without IL-33 by ChIP sequence analysis and found that new Cbfβ binding peaks appeared in ILC2s cultured with IL-33 (Fig. 5c). The GATA-3 motif was not ranked in at least the top ten motifs of the Cbfβ binding peaks specific for ILC2s stimulated with IL-33 (Fig. 5d). Furthermore, the IL-33-specific Cbfβ binding peaks were located at genes for ILC2 function, including *Il5*, *Il13*, *Lta*, *Nmur1*, and *Vipr2*, and genes for exhaustion markers, including *Il10*, *Tigit*, *Prdm1*, *Lag3*, and *Ctla4* (Fig. 5e). Examples of these GATA-3-independent Cbfβ biding peaks are shown in Fig. 6a. Thus, Cbfβ binding specific to IL-33 stimulation is independent of GATA-3 and is associated with a gene expression profile of exhausted-like ILC2s, suggesting a GATA-3-independent function of the Runx proteins in ILC2 reactivity against IL-33.

### Enhancer or repressor functions of Runx in activated ILC2s

We assessed the possibility that Runx/Cbfβ complexes bound to enhancers of gene loci related to ILC2 activity and repressors of exhaustion marker gene loci in ILC2s stimulated with IL-33. For this purpose, we determined which Cbfb binding peaks were marked by H3K27 acetylation for enhancer regions or H3K27 trimethylation for repressed gene regions in ILC2s stimulated with IL-33 (Fig. 6a, b and Supplementary Data 2). Cbfβ binding peaks overlapping with H3K27 acetylation peaks were associated with ILC2 functional genes that were downregulated in the hyporesponsive Cbfβ-deficient ILC2s (Fig. 4e). Gene ontology analysis of the genes with both Cbfβ and H3K27 acetylation peaks indicated that Cbfβ globally regulated genes involved in positive regulation of proliferation, cytokine production, cytokine-mediated signaling, leukocyte migration, and leukocyte adhesion (Fig. 6c). In contrast, the H3K27 trimethylation status of the exhaustion marker genes was variable. Among the genes listed as exhaustion markers in Fig. 4h, H3K27 trimethylation was observed in the *Il10*, *Prdm1*, and *Ctla4* loci. Since IL-10 is a good marker for exhausted-like ILC2s, we confirmed the reduced H3K27 trimethylation level in the *Il10* promoter region of *Cbfb*^f/f^ PLZF-Cre ILC2s activated by IL-33, indicating that the *Il10* locus was repressed by Runx proteins in part through H3K27 trimethylation (Fig. 6d). Collectively, Runx/Cbfβ complexes have comprehensive effects as transcription factors on the gene expression profile of exhausted-like ILC2s.

### Runx deficiency in ILC2s ameliorates allergic inflammation

**Fig. 7:**
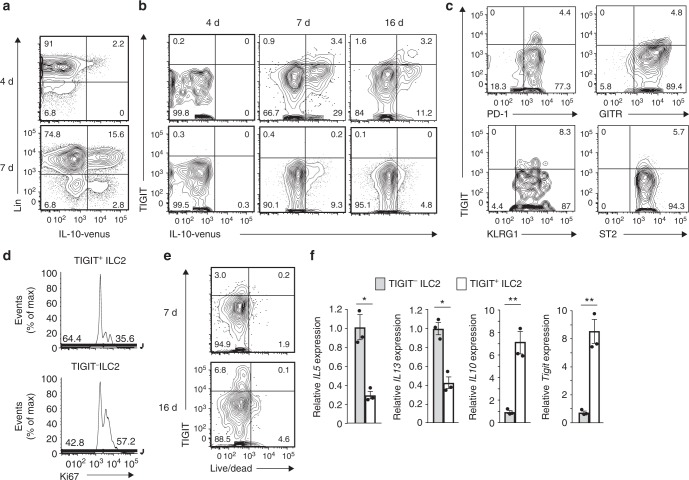
Exhausted-like ILC2s appear at the severe inflammation site. **a**–**f** IL-10-Venus mice (**a**, **b**) or C57BL/6 mice (**c**–**f**) were administered papain intranasally every 3 days for the indicated time frame. **a**, **b** Flow cytometry analysis of TIGIT and IL-10-Venus expression by lineage-negative or positive cells (**a**) and ILC2s (CD45^+^ CD3^–^ CD19^–^ NK1.1^–^ CD11b^–^ Gr1^−^ Ter119^–^ CD127^+^ CD25^+^ ST2^+^) in the bronchoalveolar lavage (BAL) fluid (**b**) on the indicated day. **c** Flow cytometry analysis of expression of the indicated molecules by ILC2s in the BAL fluid on day 7. **d** Flow cytometry analysis of Ki67 expression by TIGIT^+^ (top) or TIGIT^–^ ILC2s (bottom) in the BAL fluid on day 7. **e** Flow cytometry analysis of TIGIT expression and the Live/Dead marker by ILC2s in the BAL fluid on days 7 and 16. **f** Quantitative RT-PCR analysis of the indicated gene transcripts in TIGIT^–^ and TIGIT^+^ ILC2s in the BAL fluid on day 7. For the flow cytometry plots, the numbers indicate the percentages of cells in each quadrant (**a**–**c**, **e**). In **f**, *p < 0.05 and **p < 0.01 by Student’s *t*-test. Data are representative of at least two independent experiments (mean ± s.d. of technical triplicates collected from 18 mice in **f**)

ILC2_10_s can be found in vivo during chronic or severe inflammation^12^. To identify TIGIT^+^ IL-10^+^ ILC2s in physiological settings, we took advantage of a severe subacute asthma model with IL-10-Venus reporter mice administered a high dose of papain every three days. On day 7 after administration of three papain doses, ILC2s producing IL-10 were observed in the BAL fluid and lung (Fig. 7a, b). However, TIGIT^+^IL-10^+^ ILC2s were found only in the BAL fluid, which was the site of severe inflammation, but not in the lung. Since most TIGIT^+^ ILC2s expressed IL-10-Venus, TIGIT^+^ ILC2s can represent TIGIT^+^IL-10^+^ ILC2s. The TIGIT^+^ ILC2s in the BAL fluid are a small population of activated ILC2s that are marked by PD-1, GITR, and KLRG1 expression^15,25^ and are not inflammatory ILC2s due to normal ST2 expression (Fig. 7c)^22^. TIGIT^+^ ILC2s did not proliferate well, as determined by diminished Ki67 staining and were negative for a dead cell marker (Fig. 7d, e). The RT-PCR assay indicated lower *Il5* and *Il13* expression in the TIGIT^+^ ILC2s than in the TIGIT^–^ ILC2s. The hyporesponsive TIGIT^+^ ILC2s emerged in the lung, as well as in the BAL fluid, when the mice were treated with papain every three days for a month (Supplementary Fig. 7a). Furthermore, to examine whether the TIGIT^+^IL-10^+^ ILC2s in the BAL fluid were similar to the hyporesponsive Cbfβ-deficient ILC2s, we performed RNA sequence analysis by barcoding individual cDNA molecules from one hundred TIGIT^+^ and TIGIT^–^ ILC2s. The sample number (*n* = 3) and assay sensitivity were not sufficient to observe a significant reduction of *Il5* and *Il13* expression in the TIGIT^+^ ILC2s, although the *Il5* and *Il13* transcript levels were significantly decreased in the TIGIT^+^ ILC2s based on a sensitive RT-PCR analysis (Fig. 7f, Supplementary Fig. 8, and Supplementary Data 3). Despite these limitations, the gene set enrichment analysis showed that the signature genes of TIGIT^+^ ILC2s in the BAL fluid were enriched in the low-reactive Cbfβ-deficient ILC2s. Thus, severe allergy induced hyporesponsive TIGIT^+^ IL-10^+^ ILC2s similar to Cbfβ-deficient ILC2s.

**Fig. 8:**
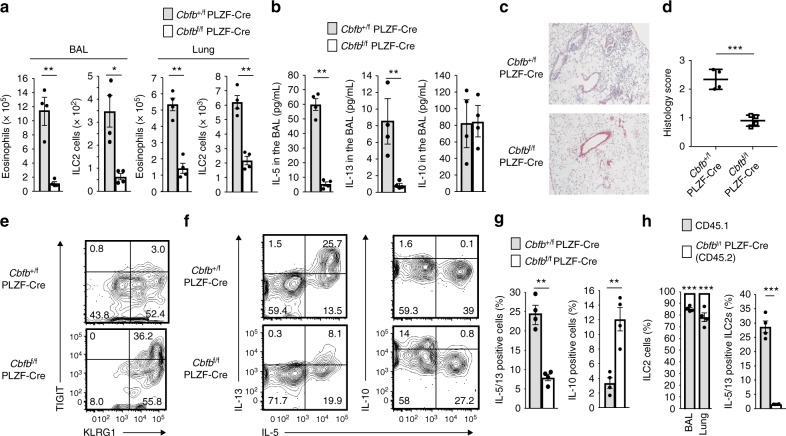
Cbfβ contributes to allergy by inhibiting the emergence of exhausted-like ILC2s. CD45.1^+^ congenic mice were adoptively transferred with bone marrow cells from either *Cbfb*^+/f^ PLZF-Cre or *Cbfb*^f/f^ PLZF-Cre mice and administered papain intranasally every 3 days three times at 12 weeks after transfer. On the day following the last papain treatment, assays were performed. **a** Absolute numbers of eosinophils (CD45^+^ CD11c^–^ Siglec F^+^) and ILC2s in the BAL fluid and the lung from the indicated recipient mice. **b** The IL-5, IL-13, and IL-10 concentrations in the BAL fluid from the indicated recipient mice. **c**, **d** HE staining (**c**) and histology scores (**d**) of the lung from the indicated recipient mice (scale bar, 100 µm). **e**, **f** Flow cytometry analysis of TIGIT, KLRG1 (**e**), IL-5, IL-13, and IL-10 (**f**) expression by ILC2s from the lung of the indicated recipient mice. **g** The frequency of ILC2s producing both IL-5 and IL-13 or IL-10 was determined by flow cytometry as in **f**. **h** CD45.1^+^/CD45.2^+^ mice were adoptively transferred with 50% CD45.1^+^ bone marrow cells and 50% CD45.2^+^ *Cbfb*^f/f^ PLZF-Cre bone marrow cells. At 12 weeks after transfer, the recipient mice were treated with papain as in **a**. Chimerism of CD45.1^+^ and CD45.2^+^ ILC2s in the BAL fluid and lung (right) and the frequency of ILC2s producing IL-5 and IL-13 of the CD45.1^+^ and CD45.2^+^ ILC2s in the lung was determined by flow cytometry (left). For the flow cytometry plots, the numbers indicate the percentages of cells in each quadrant (**e**, **f**). In **a**, **b**, **g**, **h**, *p < 0.05, **p < 0.01, and *p < 0.001 by Student’s *t*-test. Data are representative of at least two independent experiments (mean ± s.d. of four mice in **a**, **b**, **g** and of three mice in **h**)

To investigate the impact of increased exhausted-like ILC2s resulting from Cbfβ deficiency on inflammation, we created a subacute asthma model with a high dose of papain using congenic mice adoptively transferred with bone marrow cells from either *Cbfb*^+/f^ PLZF-Cre or *Cbfb*^f/f^ PLZF-Cre mice. As expected, the recipients of the *Cbfb*^f/f^ PLZF-Cre bone marrow cells had reduced eosinophils and ILC2s in the BAL fluid and lungs (Fig. 8a), which were accompanied by a reduction in IL-5 and IL-13 production in the BAL fluid (Fig. 8b), as well as less immune cell infiltration around the bronchi (Fig. 8c, d) than those of the *Cbfb*^+/f^ PLZF-Cre cell recipients. Furthermore, ILC2s without Cbfβ function in the lung generated an increased TIGIT^+^ fraction and produced less IL-5 and IL-13 but more IL-10 than the control ILC2s (Fig. 8e–g). However, the increased IL-10 from the Cbfβ-deficient ILC2s did not seem to be responsible for the reduced allergic inflammation, because the IL-10 concentration of the BAL fluid was not increased in the papain-treated recipients of the *Cbfb*^f/f^ PLZF-Cre bone marrow cells, probably due to higher IL-10 production by lineage^+^ cells than by ILC2s (Figs. 7a, 8b). Cell-intrinsic hyporeactivity of *Cbfb*^f/f^ PLZF-Cre ILC2s was also confirmed by bone marrow competition (Fig. 8h). Thus, the exhausted-like phenomenon in ILC2s was associated with decreased inflammation mediated by eosinophils.

To determine the function of Cbfβ-deficient ILC2s in the chronic allergy model, mice were continuously administered a high dose of papain every three days for one month as a severe chronic allergy model (Supplementary Fig. 7c) or a high dose of papain every three days three times followed by the same course of papain treatment after a two week cessation period as a repeated chronic allergy model (Supplementary Fig. 7d). Cbfβ-deficient ILC2s did not respond well to papain stimulation in the model mice. However, the hyporesponsive *Cbfb*^f/f^ PLZF-Cre ILC2s were associated with reduced eosinophil recruitment only after a 2nd course of papain challenge. Thus, Runx/Cbfβ complexes in ILC2s play a critical role in inflammatory responses to repeated allergen stimulation.

**Fig. 9:**
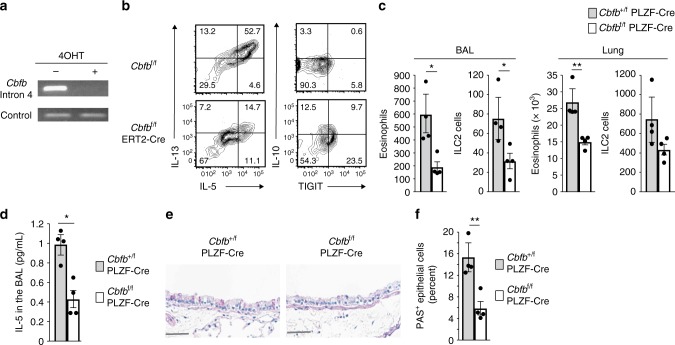
Exhausted-like hyporesponsiveness of ILC2s attenuates allergic inflammation. **a** PCR analysis of the floxed intron 4 in the *Cbfb* gene compared to the unfloxed genes (control) in *Cbfb*^f/f^ ERT2-Cre ILC2s cultured with IL-2, IL-7, and IL-33 with or without 4-hydroxytamoxifen (4OHT). **b** Flow cytometry analysis of IL-5, IL-13, IL-10, and TIGIT expression by *Cbfb*^f/f^ or *Cbfb*^f/f^ ERT2-Cre ILC2s cultured with 4OHT. **c**–**f** *Rag2*^–/–^ *Il2rg*^–/–^ mice were adoptively transferred with *Cbfb*^f/f^ or *Cbfb*^f/f^ ERT2-Cre ILC2s (1 × 10^6^ cells/mouse) prepared as in **b** and administered papain intranasally on days 0, 1, and 2. On day 3, the absolute numbers of eosinophils and ILC2s in the bronchoalveolar lavage (BAL) fluid and lung (**c**) and the IL-5 concentration in the BAL fluid (**d**) from the recipient mice were analyzed by flow cytometry and ELISA, respectively. Histology (**e**) and the frequency (**f**) of PAS-positive lung epithelial cells in the indicated recipients. In **c**, **d**, **f**, *p *<* 0.05 and **p < 0.01 by Student’s *t*-test. Data are representative of at least two independent experiments (mean ± s.d. of four mice in **c**, **d**, **f**)

To further determine the effect of Cbfβ deficiency in ILC2s on allergic inflammation, we adoptively transferred Cbfβ-deficient ILC2s prepared by Cbfβ deletion during in vitro culture of lung ILC2s into *Rag2*^–/–^ *Il2rg*^–/–^ mice lacking both acquired immunity and innate lymphoid cells. Prior to the adoptive transfer, we confirmed acquisition of an exhausted-like phenotype including high IL-10 and TIGIT expression with low IL-5 and IL-13 production due to Cre-mediated conversion of the floxed allele (Fig. 9a, b). The *Rag2*^–/–^ *Il2rg*^–/–^ recipients were intranasally administered papain for 3 consecutive days. Analysis of these mice on day 3 revealed that the transferred Cbfβ-deficient ILC2s were less capable of recruiting eosinophils to the bronchoalveolar space and the lung (Fig. 9c), with a decreased amount of IL-5 in the BAL fluid and increased epithelial damage (Fig. 9d–f). These data indicate that Cbfβ is required for the ability of ILC2s to trigger and extend allergic inflammation and to prevent them from falling into an exhausted-like state in inflamed tissues.

## Discussion

We have shown here that Runx/Cbfβ complexes suppress the basal activity of ILC2s, which can recruit eosinophils through IL-5 production under steady-state conditions. However, under allergic conditions, Runx/Cbfβ complexes support the ability of ILC2s to exert their helper functions for type 2 immunity. Diminished activity of ILC2s lacking Runx under allergic inflammatory conditions was accompanied by increased expression of T cell exhaustion markers, such as IL-10 and TIGIT. Mechanistically, Runx/Cbfβ complexes contribute to epigenetic modification for a gene expression profile of the exhausted-like ILC2s. These TIGIT^+^ IL-10^+^ ILC2s were identified even in mice with allergen-induced subacute or chronic inflammation. Finally, transferred ILC2s with exhausted-like characteristics lacked an appropriate capacity for allergic immune responses in vivo. Thus, our results revealed an essential regulation of ILC2 function by Runx proteins and an accelerated emergence of exhausted-like ILC2s in the absence of Runx/Cbfb complexes.

We used PLZF-Cre mice to induce dysfunction of Cbfβ in ILC2s. We obtained a Cbfβ deletion that was rather specific to ILC subsets in *Cbfb*^f/f^ PLZF-Cre mice, although PLZF-Cre is expressed in unknown progenitor cells for most haematopoietic cells. We cannot completely deny the possibility that any small population of haematopoietic cells may be affected in the *Cbfb*^f/f^ PLZF-Cre mice. Therefore, using PLZF-Cre mice for an ILC study is quite risky and requires precise examination of the deletion effect on the haematopoietic populations, as described in our paper.

Since both TIGIT and IL-10 are induced by IL-33^12^, a strong IL-33 signal should be required for the generation of TIGIT^+^ IL-10^+^ ILC2s. The hyporesponsive TIGIT^+^ IL-10^+^ ILC2s are apparently part of the activated ILC2s, because they express a series of activation markers, including PD-1 and KLRG1, which are also known inhibitory molecules. T cells become exhausted through interaction of their inhibitory receptors, such as CTLA-4 and PD-1, with cognate ligands on other cells^14^. Therefore, we can reasonably assume that the low reactivity of TIGIT^+^ IL-10^+^ ILC2s results from accumulated signals through those inhibitory molecules. Although we did not test the function of the individual inhibitory molecules on the TIGIT^+^ IL-10^+^ ILC2s, we clearly showed that the emergence of hyporeactive TIGIT^+^ IL-10^+^ ILC2s was negatively regulated by Runx/Cbfβ complexes.

Chronic or severe inflammation is required to induce TIGIT^+^ IL-10^+^ ILC2s. If ILC2s have functional Runx proteins, then hyporesponsive TIGIT^+^ IL-10^+^ ILC2s are rare even after continuous inhalation of papain for one month. Furthermore, *Cbfb*^f/f^ PLZF-Cre ILC2s with low reactivity were not associated with attenuation of chronic allergy. These data indicate that TIGIT^+^ IL-10^+^ ILC2s and ILC2s themselves play a limited role in the pathology of chronic inflammation. However, hyporesponsive ILC2s lacking Cbfβ protect the host from exaggerated allergic inflammation when repeatedly treated with allergen. Therefore, regulation of ILC2 functions by Runx/Cbfb complexes is critical for the pathogenesis of acute exacerbation of chronic allergy.

ILC2_10_s are identified by low *Tnf*, *Lta*, *Il2*, *Retnla*, and *Ccl1* expression and high *Il10* and *Id3* expression compared to the expression levels of the non-IL-10 producers^12^. Cbfβ-deficient ILC2s are somewhat similar to ILC2_10_s in that *Tnf*, *Lta, Retnla* and *Ccl1* expression is reduced in both cell types and IL-10 production is increased. However, Cbfβ ablation resulted in more global defects in ILC2 function, because ILC2s lacking Cbfβ poorly expressed the main effector cytokines, such as *Il5* and *Il13*, whereas ILC2_10_s produce large amounts of IL-5 and IL-13^12^. Furthermore, the expression of T cell exhaustion markers is not increased on ILC2_10_s, whereas the high *Id3* expression observed in ILC2_10_s is not induced in Cbfβ-deficient ILC2s. Thus, the hyporesponsive ILC2s lacking Cbfβ seem to be different from ILC2_10_s.

Immunosenescence is another hyporesponsive state of immune cells that results from the effects of ageing. Cell cycle arrest related to telomere shortening or DNA damage is thought to be a common feature of cellular senescence^26^. Runx1 is involved in age-related changes in haematopoietic stem cells^27^. The regenerative capacity of HSCs declines following conditional ablation of Runx1 after an initial expansion^28^. In our study, we utilized a subacute or repeated allergy model to induce ILC2 hyporesponsiveness. Testing whether aging could be a trigger for senescent ILC2s and whether the absence of Runx function was involved in ILC2 senescence would be fascinating.

The concept of exhausted-like ILC2s provides a better understanding of normal ILC2 physiology. Many mouse models have successfully demonstrated the importance of ILC2s during the acute phase of an allergic response^29^. In addition, an important role of ILC2s in worsening of recurrent allergy has been suggested. ILC2s can also be trained by the initial allergic stimulation, resulting in production of higher T_H_2 cytokine levels after a second challenge^13^. The immune system always has delicate balancing mechanisms to maintain homeostasis. Exhausted-like hyporesponsiveness is one mechanism by which immune cells are prevented from overactivation and continuous production of harmful inflammatory cytokines. Therefore, ILC2s may possess a capacity to reduce their activity similar to that of exhausted T cells after chronic exposure to allergic stimuli.

The frequency and activity of ILC2s are both increased in patients with chronic type 2 inflammation, including asthma and atopic dermatitis and are also associated with the disease severity^30–32^. Given a possible role of chronic inflammation in tissues in providing environmental cues to induce low reactive ILC2s, an examination of whether hyporesponsive ILC2s are present in and beneficial for patients with chronic allergy will be important, especially in cases of acute disease exacerbation. Our study in mice revealed that attenuated Runx function accelerated the differentiation of ILC2s towards a hyporesponsive state. Further understanding of the molecular switch triggering exhausted-like ILC2s may provide a basis for the development of therapeutic targets to suppress allergic immune responses.

## Methods

### Mice

All mice were maintained at the RIKEN Center for Integrative Medical Sciences. The animal protocol was approved by the Institutional Animal Care and Use Committee of RIKEN Yokohama Branch. C57BL/6 mice and congenic CD45.1^+^ mice were obtained from the National Cancer Institute. IL-10-Venus reporter mice were kindly provided by Dr Kiyoshi Takeda at Osaka University. The *Cbfb*^f/f^ (Stock No. 008765), *Runx1*^f/f^ (Stock No. 008772), *Runx3*^f/f^ (Stock No. 008773), PLZF-Cre (Stock No. 024529), and ERT2-Cre mice (Stock No. 008463) were all obtained from Jackson Laboratories. All Cre-expressing mice were heterozygous.

### Cell preparation

Cells were isolated from the spleen, liver, BAL fluid, lung, and small intestine as previously reported^17^. The spleen and liver were dissected and smashed through a 70 µm strainer. Lymphocytes from the liver were resuspended in 40% Percoll and centrifuged at 2000 rpm at room temperature for 20 min. Cells at the bottom of the tube were collected for flow cytometry after red blood cell (RBC) lysis. BAL fluid was obtained by infusion of PBS through a catheter. The dissected lung was cut into small pieces, chopped with a razor blade, and incubated in 8 mL of digestion buffer containing RPMI medium with 2% FBS, 0.5 mg per mL of Collagenase IV (Sigma, C5138), and 0.05 mg per mL of DNase (Wako, 043-26773) at 200 rpm and 37 °C for 45 min. Digested cells were smashed through a 70 µm strainer and used for flow cytometry and cell culture after RBC lysis. Lung lymphocytes were further purified with a 40 and 80% Percoll gradient for sorting. Intraepithelial lymphocytes (IELs) and LPLs were isolated from the small intestine. After removing feces and Peyer’s patches, the small intestine was incubated in 20 mL of RPMI medium with 2% FBS and 5 mM EDTA at 200 rpm and 37 °C for 20 min. After vigorous vortexing, floating cells were collected as the IELs. The remaining tissues were cut into small pieces, chopped with a razor blade, and incubated in 20 mL of the same digestion buffer at 200 rpm and 37 °C for 30 min. Digested cells containing LPLs or the IELs collected above were purified with a 40 and 80% Percoll gradient for flow cytometry, culture and sorting. Lymphocytes from the lung and intestine were cultured with GoldiPlug without any stimulation to assess ex vivo production of IL-5 and IL-13 or with PMA (50 ng per mL) and Ionomycin (0.5 µg per mL) to analyze cytokine production by ILC2s from mice treated with papain for 4 h. ILC2s were sorted from the lung with the FACSAria (BD Biosciences). Five hundred ILC2s were cultured with IL-2 (10 ng per mL), IL-33 (10 ng per mL), and with/without IL-7 (10 ng per mL). For inducible deletion of *Cbfb*, 1 µM of 4-hydroxytamoxifen (4OHT) was added to the cell culture for 4 days.

### Antibodies and flow cytometry

Cells were blocked with an anti-CD16/32 antibody (2.4g2) first and then stained with Fixable Viability Dye eFluor 506 (eBioscience) for detection of dead cells before staining the cell surfaces. The antibodies used for flow cytometry are all listed in Supplementary Table 1. The Cytofix/Cytoperm Buffer Set (BD Biosciences) and Foxp3/Transcription Factor Staining Buffer Set (eBioscience) were used for staining of intracellular cytokines and transcription factors, respectively, according to the manufacturers’ protocols. Data were acquired on a FACSCanto II (BD Biosciences) and analyzed with the FlowJo software (TreeStar). Gating and sorting strategies were described in Supplementary Fig. 9.

### PCR and RT-PCR

Cells were directly sorted into DNA extraction buffer (0.1 M Tris-HCL pH 7.5, 0.05 M EDTA pH 8.0, and 1.25% SDS) for DNA or TRIzol (Thermo Fisher Scientific) for RNA by the FACSAria. DNA was purified from the cell lysates with Phenol-Chloroform Isoamyl Alcohol, followed by ethanol precipitation. RNA was purified, and cDNA was synthesized with the PrimeScript™ 1st Strand cDNA Synthesis Kit (Takara) following the manufacturer’s protocol. Then, the mRNA transcripts were quantified with TB Green™ Premix Ex Taq™ II (Takara). The relative expression of the indicated transcripts was calculated by the 2^−ΔCt^ method and normalized to *Actb* expression. The copy numbers of *Cbfb* transcripts with or without a floxed region were calculated by quantitative RT-PCR. The specific primers are listed in Supplementary Table 2.

### Bone marrow competition

Recipient CD45.1^+^ or CD45.1^+^/CD45.2^+^ congenic mice were lethally irradiated at 950 rad and reconstituted with 5 × 10^6^ bone marrow cells from *Cbfb*^+/f^ PLZF-Cre (CD45.2^+^) mice or 5 × 10^6^ CD45.1^+^ bone marrow cells from *Cbfb*^f/f^ PLZF-Cre (CD45.2^+^) mice. At 8–12 weeks after transfer, the presence of the indicated cells was analyzed in the spleen, bone marrow, lung, liver, and small intestine.

### Papain-induced asthma model

After irradiation at 950 rad, CD45.1^+^ congenic mice were transferred with 1 × 10^7^ bone marrow cells from either *Cbfb*^+/f^ PLZF-Cre or *Cbfb*^f/f^ PLZF-Cre mice. At 12 weeks after transfer, 100 µg of papain in 50 µL of sterile PBS was intranasally administered to the recipient mice every 3 days. On day 7 or 14, a 20-G catheter was inserted into the trachea, and 1 mL of PBS was infused into the lung through the catheter and aspirated 3 times. The first BAL fluid sample was used to detect IL-5, IL-10, and IL-13 by ELISA. Cells in the whole BAL fluid were analyzed by flow cytometry. After making a knot at the left main bronchus, the left lung was removed to isolate cells for flow cytometry. The right lung was intratracheally infused with 1 mL of 10% formalin, removed, and incubated in 10% formalin at 4 °C overnight to make paraffin blocks. HE and PAS staining were performed as previously reported^17^. To expand ILC2s from the lungs of the *Cbfb*^f/f^ and *Cbfb*^f/f^ ERT2-Cre mice, ILC2s were cultured in RPMI medium with 10% FBS, 55 µM 2ME, 1% Pen Strep, 10 ng per mL of IL-2 (Peprotech), and 10 ng per mL of IL-7 (Peprotech) for 2–4 weeks. For adoptive transfer of *Cbfb*^f/f^ or *Cbfb*^f/f^ ERT2-Cre ILC2s without in vitro tamoxifen treatment into *Rag2*^–/–^ *Il2rg*^–/–^ mice, 1 × 10^6^ cells were intravenously injected, and the mice were treated with tamoxifen as described below. To delete *Cbfb* and stimulate ILC2s in vitro, 1 µM of 4-hydroxytamoxifen (4OHT) was added to the culture with 10 ng per mL of IL-33. At day 4 after 4OHT treatment, 4OHT was removed from the cell culture. At day 7 after 4OHT treatment, the ILC2s were collected and injected into *Rag2*^–/–^ *Il2rg*^–/–^ mice (1 × 10^6^ cells/mouse). Then, 100 µg of papain in 50 µL of PBS was intranasally administered to the *Rag2*^–/–^ *Il2rg*^–/–^ mice on days 0, 1, and 2. On day 3, the BAL fluid and lung were collected as described above.

### Histology score

Lung injury was scored as previously described^33^. Briefly, scores were given as follows: grade 1, a few inflammatory cells around the bronchus; grade 2, a layer one cell deep around the bronchus; grade 3, a layer two to four cells deep around the bronchus; and grade 4, a layer of more than four cells deep surrounded the bronchus. Six airways per section were randomly selected for scoring. PAS-positive lung epithelial cells were counted at a magnification of ×400 when at least one clear PAS-positive cell was found.

### Inducible deletion of Cbfβ, Runx1 and Runx3 in vivo

*Cbfb*^f/f^ ERT2-Cre, *Runx1*^f/f^ ERT2-Cre, *Runx3*^f/f^ ERT2-Cre, *Runx1*^f/f^ *Runx3*^f/f^ ERT2-Cre, and *Rag2*^–/–^ *Il2rg*^–/–^ recipient mice transferred with *Cbfb*^f/f^ or *Cbfb*^f/f^ ERT2-Cre ILC2s were orally administered 4 mg of tamoxifen (Sigma) in 200 µL of corn oil (Wako) for 5 consecutive days. At 3-4 weeks after tamoxifen treatment, ILC2s were isolated from the small intestine LPLs for flow cytometry and short-term culture to analyze ex vivo IL-5 production as described above. Eosinophils in the BAL fluid and PAS-positive lung epithelial cells of the *Rag2*^–/–^ *Il2rg*^–/–^ recipient mice were analyzed by flow cytometry and histology, respectively.

### RNA sequencing

RNA was extracted from ILC2s sorted from the lungs of *Cbfb*^+/f^ PLZF-Cre or *Cbfb*^f/f^ PLZF-Cre mice and from lung ILC2s cultured with 10 ng per mL of IL-2 and 10 ng per mL of IL-33 in vitro for 4 days using TRIzol (Qiagen), followed by the RNeasy micro kit (Qiagen). Sequencing libraries were prepared with a SMARTer Pico kit (Clontech). Single end 50 bp reads were obtained by an Illumina HiSeq 1500. The reads were mapped and analyzed with TopHat v2.1.0 and Cufflinks 2.2.1. Heat maps were generated from the z-score by Morpheus (Broad Institute). Gene set ontology analysis (Broad Institute) was performed with a gene set that was positively or negatively regulated by GATA-3 and gene expression data (RPKM) from the *Cbfb*^+/f^ PLZF-Cre and *Cbfb*^f/f^ PLZF-Cre ILC2s; a gene set for a CD8^+^ T cell exhaustion signature was previously described^34^, and gene expression data were obtained from the *Cbfb*^+/f^ PLZF-Cre and *Cbfb*^f/f^ PLZF-Cre ILC2s. EdgeR was used to calculate differentially expressed genes and draw the MA plot.

### Chromatin immunoprecipitation sequencing

ILC2s were sorted from the lungs of C57BL/6 mice and expanded in vitro in RPMI medium with 10% FBS, 55 µM 2ME, 1% Pen Strep, 10 ng per mL of IL-2 (Peprotech), 10 ng per mL of IL-7 (Peprotech), and 10 ng per mL of IL-33 (Peprotech) for 4 weeks to expand the cells. Then, the ILC2s were cultured with or without IL-33 for 1–2 weeks, and 1.5 × 10^7^ ILC2s were collected per ChIP-seq sample. For ChIP followed by qPCR, lung ILC2s from *Cbfb*^+/f^ PLZF-Cre and *Cbfb*^f/f^ PLZF-Cre mice were cultured with IL-2 and IL-7 for 3 weeks and stimulated with IL-2, IL-7, and IL-33 for one week. We followed the ChIP-seq protocol described elsewhere^17,35^. Briefly, after 10 min of fixation in 1% paraformaldehyde at room temperature, the reaction was stopped by a glycine solution (final concentration of 0.15 M), and the cells were lysed in lysis buffer 1 (50 mM HEPES pH 7.5, 140 mM NaCl, 1 mM EDTA, 10% glycerol, 0.5% NP40, and 0.25% Triton X-100) with cOmplete protease inhibitor cocktail tablets (Roche). The nuclei were pelleted and then washed with lysis buffer 2 (10 mM Tris-HCl pH 8.0, 200 mM NaCl, 1 mM EDTA and 0.5 mM EGTA) with the cOmplete protease inhibitor. The nuclei were resuspended in lysis buffer 3 (10 mM Tris-HCl pH 8.0, 100 mM NaCl, 1 mM EDTA, 0.5 mM EGTA, 0.1% sodium deoxycholate and 0.5% N-laurylsarcosine sodium salt) and sonicated using the model XL2000 ultrasonic cell disruptor (MICROSON). The fragmented chromatin was immunoprecipitated with an anti-Cbfβ^36^, anti-H3K27 acetylation (D5E4, Cell Signaling), anti-H3K27 trimethylation (ab6002, Abcam), anti-GATA-3 (L50-823, BD), anti-Runx1 (ab23980, Abcam), or anti-Runx3 (D6E2, Cell Signaling) antibody. After reverse cross-linking and purification steps, the ChIP’d DNA was re-sonicated with the Covaris S220. Libraries were created from the DNA with the NEBNext ChIP-seq Library Prep Master Mix set for Illumina kit (NEB) and sequenced with the Illumina HiSeq 1500. The sequences were mapped to the mouse genome using Bowtie 2. The peaks were called with the MACS2 programme or HOMER with default parameters. Visualization of binding traces and motif analysis were performed by HOMER. Differential binding of Cbfβ in ILC2s cultured with IL-2/IL-7/IL-33 and with IL-2/IL-7 was calculated by ChIPpeakAnno and HOMER. ChIPpeakAnno and HOMER were used to annotate genes with Cbfβ binding unique to IL-2/IL-7/IL-33 stimulation. ChIPpeakAnno was also used to annotate genes with Cbfb binding peaks marked by H3K27 acetylation near their coding regions. GO enrichment analysis was performed with 200 genes (logFC>1.5 and logCPM>2) using ClusterPlofiler.

### Retroviral transduction

C57BL/6 bone marrow cells were transduced with a pMSCV-Thy1.1 retroviral vector encoding Runx3 as described elsewhere^37^. Lung ILC2s were cultured with 10 ng per mL of IL-2, 10 ng per mL of IL-7, and 10 ng per mL of IL-33 in vitro. The ILC2s were transduced with a pMSCV-IRES-Thy1.1 with either GATA-3 or Runx3 and then cultured for another week. GoldiPlug was added for the last four hours, and IL-5 and IL-13 production by the transduced or untransduced cells was analyzed by flow cytometry.

### Statistical analysis

Data were analyzed by the two-tailed Student’s *t*-test with or without Welch’s correction. *P* values < 0.05 were considered statistically significant.

### Reporting summary

Further information on experimental design is available in the Nature Research Reporting Summary linked to this article.

## Supplementary information

Supplementary Information

Description of Additional Supplementary Files

Supplementary Data 1

Supplementary Data 2

Supplementary Data 3

Reporting Summary

## Data Availability

RNA sequence and ChIP sequence data have been deposited in the Gene Expression Omnibus at NCBI under primary accession code GSE111871. All other data are available from the authors upon reasonable requests.
