# Author Correction: Runx/Cbfβ complexes protect group 2 innate lymphoid cells from exhausted-like hyporesponsiveness during allergic airway inflammation

**DOI:** 10.1038/s41467-019-08932-5

**Published:** 2019-03-01

**Authors:** Chizuko Miyamoto, Satoshi Kojo, Motoi Yamashita, Kazuyo Moro, Georges Lacaud, Katsuyuki Shiroguchi, Ichiro Taniuchi, Takashi Ebihara

**Affiliations:** 1 Laboratory for Transcriptional Regulation, RIKEN Center for Integrative Medical Sciences (IMS), 1-7-22 Suehiro-cho, Tsurumi-ku, Yokohama, 230-0045 Japan; 2 Laboratory for Innate Immune Systems, RIKEN Center for Integrative Medical Sciences (IMS), 1-7-22 Suehiro-cho, Tsurumi-ku, Yokohama 230-0045 Japan; 3 Stem Cell Biology Group, Cancer Research UK Manchester Institute, The University of Manchester, Wilmslow Road, Manchester, M20 4BX UK; 4 Laboratory for Immunogenetics, RIKEN Center for Integrative Medical Sciences (IMS), 1-7-22 Suehiro-cho, Tsurumi-ku, Yokohama 230-0045 Japan; 5 Laboratory for Prediction of Cell Systems Dynamics, RIKEN Center for Biosystems Dynamics Research (BDR), Suita, Osaka, 565-0874 Japan; 6 JST PRESTO, 332-0012 Kawaguchi, Japan

Correction to: *Nature Communications*; 10.1038/s41467-019-08365-0; published online 25 January 2019

The original version of this Article contained an error in the author affiliations.

Affiliation 5 incorrectly read ‘Laboratory for Prediction of Cell Systems Dynamics, RIKEN Center for Biosystems Dynamics Research (BDR), Suite, Hyogo 565-0874, Japan.’ This has now been corrected in both the PDF and HTML versions of the Article.

