# Supplementary material for: Runx/Cbfβ complexes protect group 2 innate lymphoid cells from exhausted-like hyporesponsiveness during allergic airway inflammation
Source: Nat Commun. 2019 Jan 25;10:447. doi: 10.1038/s41467-019-08365-0 (PMC6347616; doi:10.1038/s41467-019-08365-0)
Supplement: Supplementary file 1 — Supplementary Information [file 41467_2019_8365_MOESM1_ESM.pdf]

Runx/Cbfβ complexes protect group 2 innate lymphoid cells from exhausted-like hyporesponsiveness during allergic airway inflammation

Miyamoto C, et al.

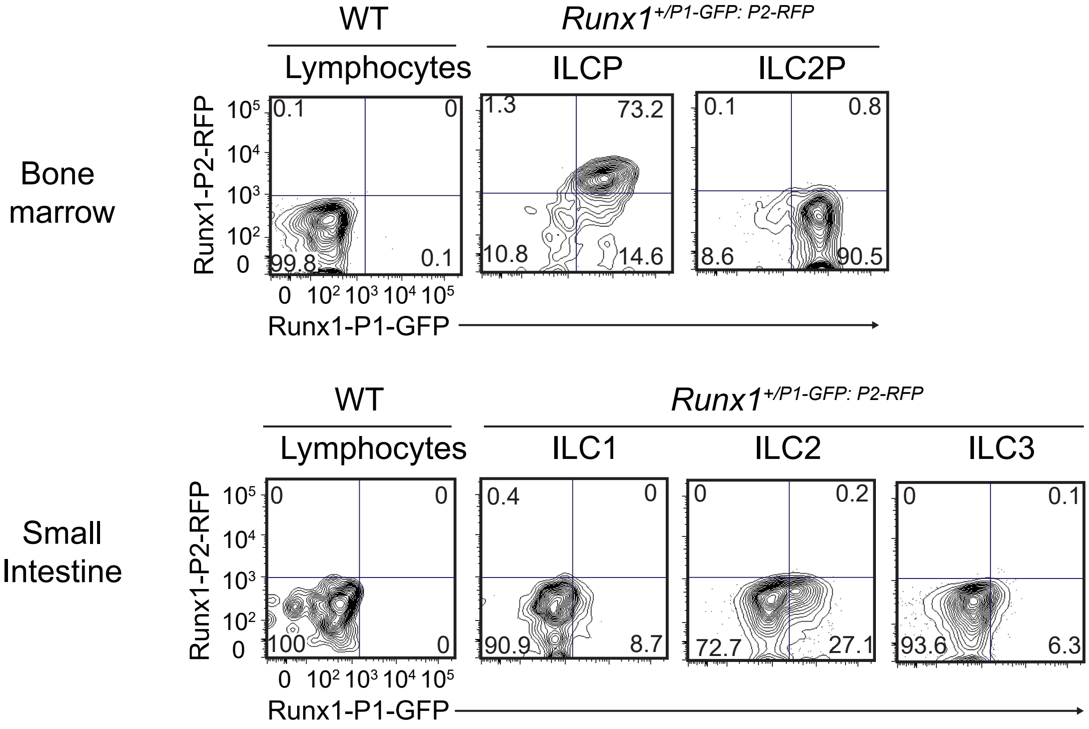

Supplementary Figure 1. Runx1 expression by ILC progenitors and ILCs. Flow cytometry analysis of the Runx1-GFP expression from the P1 promotor and the Runx1-RFP expression from the P2 promotor by ILCPs ($CD45^+$ Lin^- $cKit^+$ $CD127^+$ $\alpha4\beta7^+$ $PD\text{-}1^+$) and ILC2Ps ($CD45^+$ Lin^- $CD127^+$ $\alpha4\beta7^+$ $Flt3^-$ $CD25^+$) in the bone marrow, by ILC1s ($CD45^+$ $CD3^-$ $CD19^-$ $NK1.1^+$ $NKp46^+$ $CD127^+$), ILC2s ($CD45^+$ $CD3^-$ $CD19^-$ $NK1.1^-$ $CD127^+$ $KLRG1^+$) and ILC3s ($CD45^+$ $CD3^-$ $CD19^-$ $NK1.1^-$ $KLRG1^-$ $CD127^+$) in the small intestine, and by lymphocytes in the indicated tissues of wild type mice. Numbers indicate percent cells in each quadrant. Data are representative of at least two independent experiments.

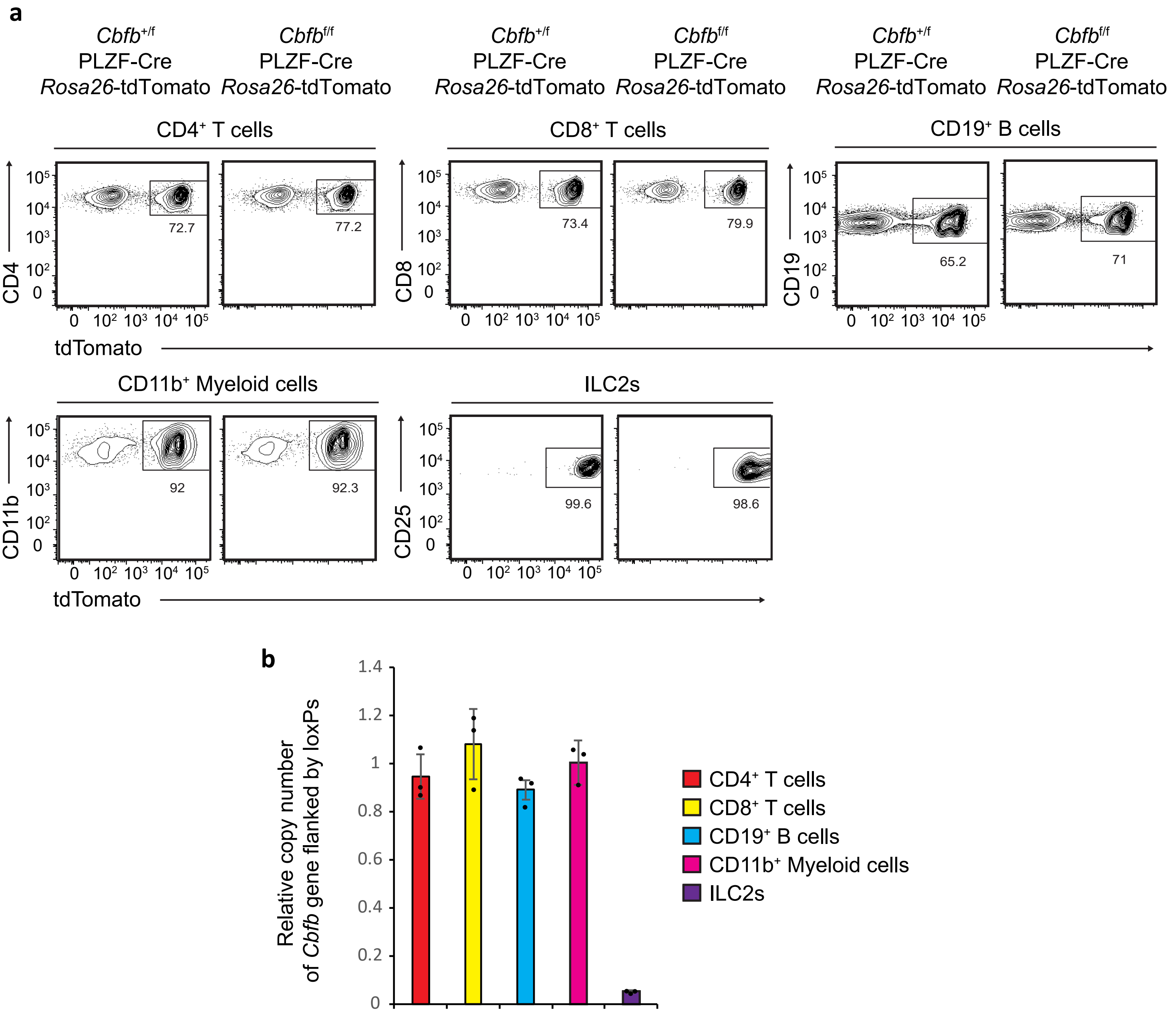

Supplementary Figure 2. *Cbfb* locus flanked by loxPs is well conserved in the major hematopoietic cells fate-mapped by PLZF expression. **a** Flow cytometry analysis of the tdTomato expression by CD4$^+$ T cells (CD3$^+$ CD4$^+$), CD8$^+$ T cells (CD3$^+$ CD8$^+$), CD19$^+$ B cells (CD3$^-$ CD19$^+$), and CD11b$^+$ myeloid cells (CD11b$^+$ NK1.1$^-$) in the spleen; and by ILC2s (CD45$^+$ CD3$^-$ CD19$^-$ NK1.1$^-$ CD127$^+$ ST2$^+$ CD25$^+$) in the lung of the indicated mice. **b** Quantitative PCR analysis for relative copy number of *Cbfb* gene flanked by loxPs in the indicated tdTomato$^+$ cells of *Cbfb*$^{f/f}$ PLZF-Cre *Rosa26*-tdTomato mice compared to those in the tdTomato$^-$ cells of *Cbfb*$^{f/f}$ PLZF-Cre *Rosa26*-tdTomato mice for CD4$^+$ T cells, CD8 T$^+$ cells, CD19$^+$ B cells, and CD11b$^+$ myeloid cells, and to tdTomato$^+$ ILC2s of *Cbfb*$^{+/f}$ PLZF-Cre *Rosa26*-tdTomato mice for lung ILC2 cells. Numbers indicate percent cells in each box. Data are representative of at least two independent experiments (mean ± s.d. of three biological replicates in **b**).

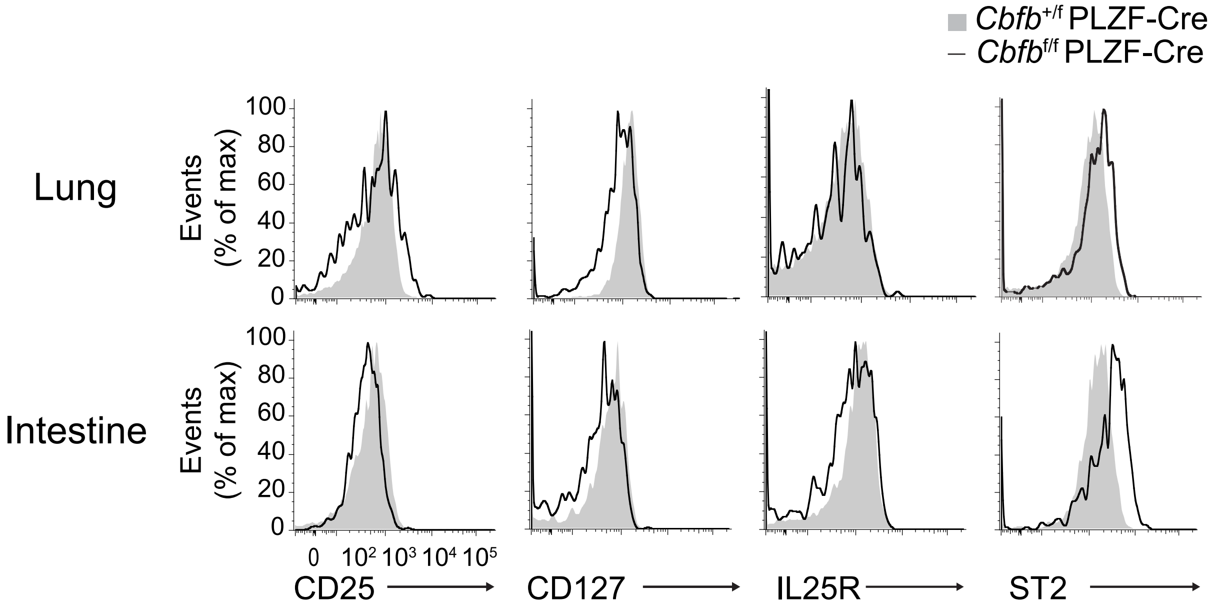

Supplementary Figure 3. Expression of cytokine receptors by Cbfβ-deficient ILC2s. Flow cytometry analysis of the indicated proteins on ILC2s (CD45$^+$ CD3$^-$ CD19$^-$ CD127$^+$ GATA-3^{Hi}) from lung (top) and intestine (bottom) of *Cbfb*$^{+/f}$ PLZF-Cre or *Cbfb*$^{f/f}$ PLZF-Cre mice. Data are representative of at least two independent experiments.

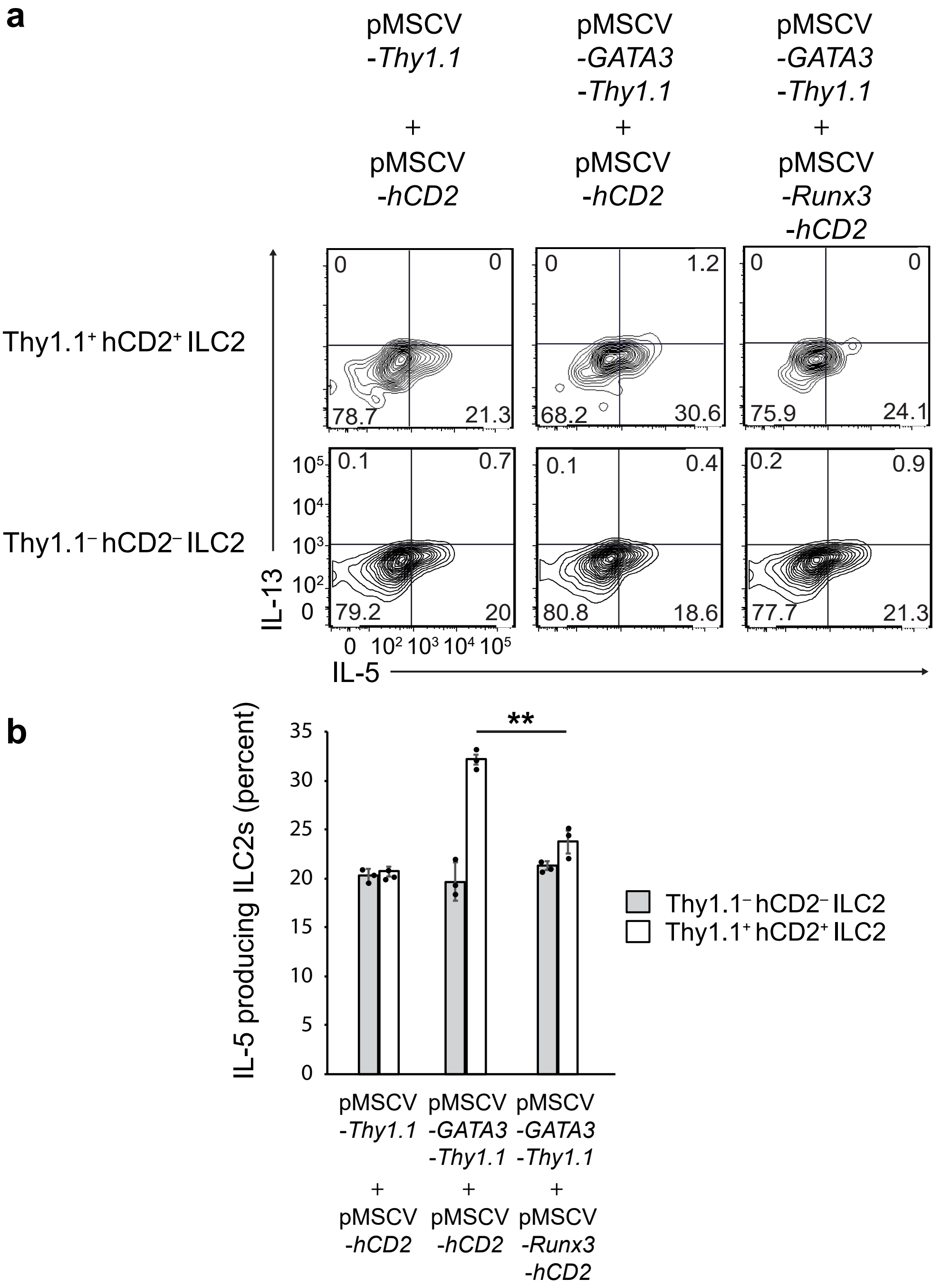

Supplementary Figure 4. Overexpression of GATA-3 ehnaces IL-5 porduction which is inhibited by Runx3. Host mice (CD45.1$^+$) were lethally irradiated and reconstituted with CD45.2$^+$ bone marrow cells retrovirally transduced with the indicated vectors. Thy1.1 and hCD2 are surrogate markers for transduction. **a** Flow cytometry analyzing the expression of IL-5 and IL-13 by transduced Thy1.1$^+$ hCD2$^+$ (top) or undtansduced Thy1.1$^-$ hCD2$^-$ (bottom) donor CD45.2$^+$ ILC2s (CD45$^+$ CD3$^-$ CD19$^-$ CD127$^+$ CD25$^+$ KLRG1$^+$) from the lung of the host mice. **b** Frequency of IL-5 producing cells in the indicated ILC2s, determined by flowcytometry as in **a**. Numbers indicate percent cells in each quadrant. In **b**, ***p* < 0.01 by Student's t-test. Data are representative of at least two independent experiments.

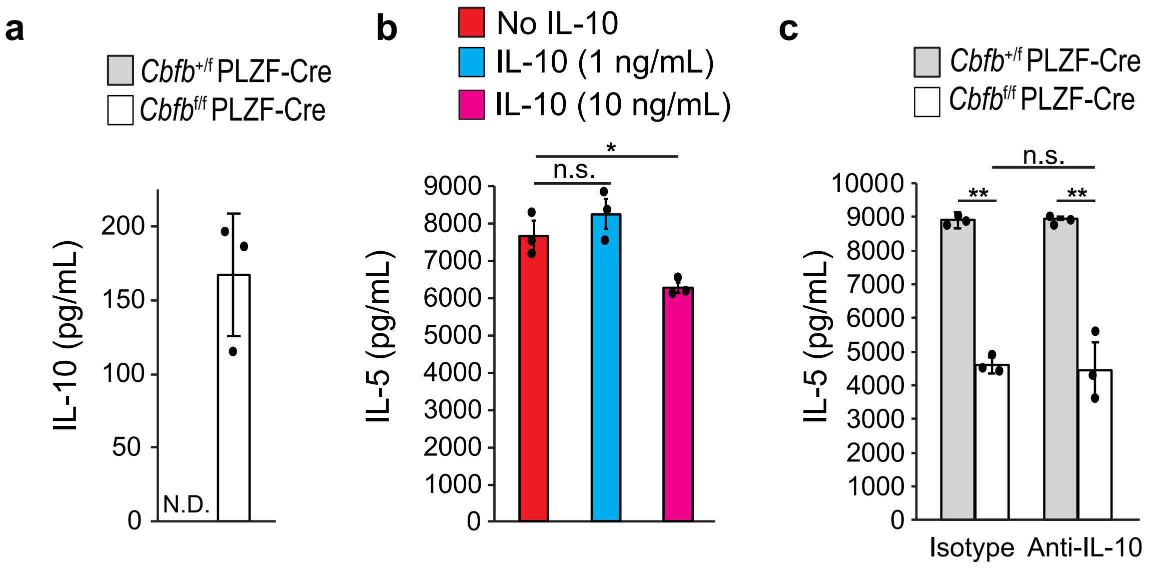

Supplementary Figure 5. IL-10 is not responsible for low reactivity of Cbfβ-deficient ILC2s. Lung ILC2s (500 cells/well) of the indicated mice were cultured with IL-2 (10 ng/mL) and IL-33 (10 ng/mL) with or without indicated reagents for 4 days. **a** Concentration of IL-10 in the culture supernatant from the lung ILC2s of the indicated mice, analyzed by ELISA. **b** Concentration of IL-5 in the culture supernatant from the C57BL/6 lung ILC2s with the indicated concentration of IL-10, analyzed by ELISA. **c** Concentration of IL-5 in the culture supernatant from the indicated mouse ILC2s with isotype control antibody and anti-IL-10 neutralizaing antibody (5 ug/mL), analyzed by ELISA. In **b** and **c**, *$p < 0.05$ and **$p < 0.01$ by Student's t-test. Data are representative of at least two independent experiments [mean ± s.d. of technical triplicates collected from three mice (a, b, c)].

a **b**

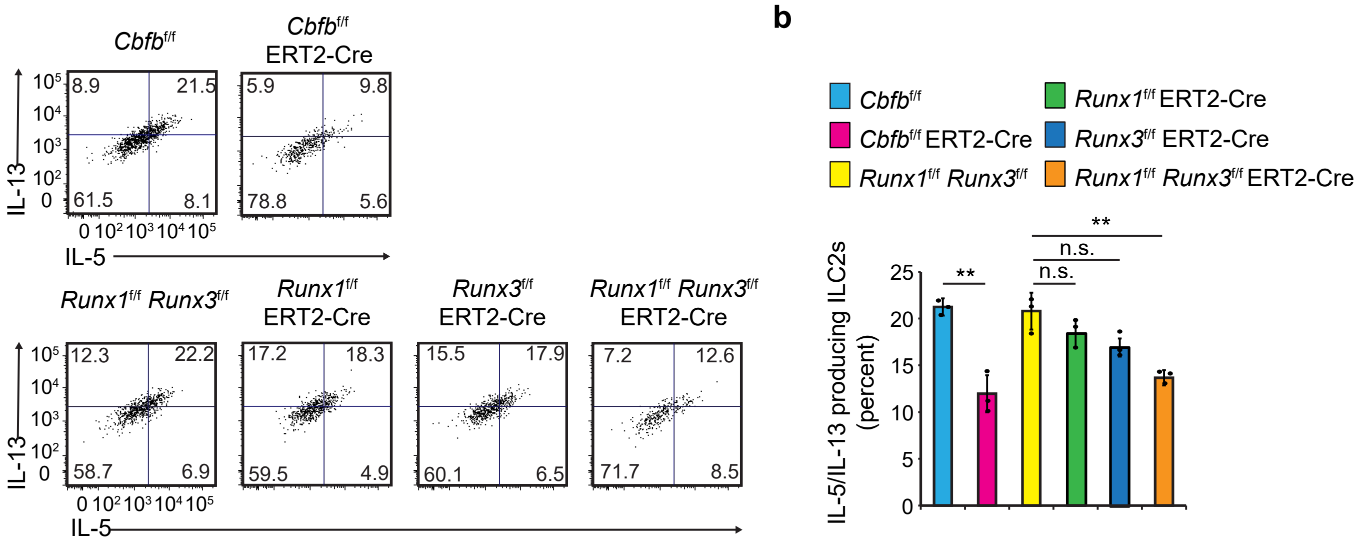

c **d**

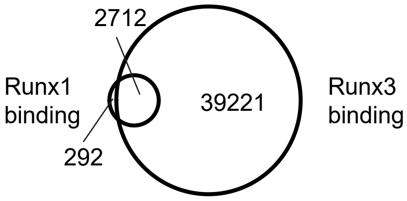

Runx1			Runx3		
TF	Motif	p-value	TF	Motif	p-value
Runx	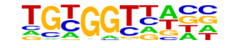	1e-351	Runx	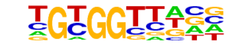	1e-6680
Ets	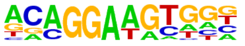	1e-307	Ets	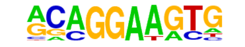	1e-2933
GATA3	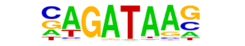	1e-146	CTCFL	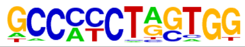	1e-2420
AP1	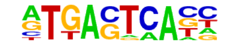	1e-146	AP1	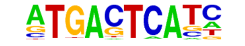	1e-2045
SP1	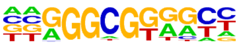	1e-92	GATA3	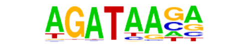	1e-897

Supplementary Figure 6. Function of Runx1 and Runx3 for ILC2 reactivity against IL-33. **a** Flow cytometry analyzing production of IL-5 and IL-13 by ILC2 from small intestine of *Cbfb*f/f ERT2-Cre, *Runx1*f/f ERT2-Cre, *Runx3*f/f ERT2-Cre, and *Runx1*f/f *Runx3*f/f ERT2-Cre mice treated with tamoxifen in responce to IL-2 and IL-33. **b** Frequency of IL-5/IL-13-producing cells in the indicated ILC2s, cultured and determined by flow cytometry as in **a**. **c** Venn graph showing number of Runx1 binding peaks (left) and Runx3 binding peaks (right) in ILC2s cultured with IL-2, IL-7, and IL-33 *in vitro,* determined by ChIP sequence analysis. **d** Motif analysis of Runx1 or Runx3 binding peaks shown in **c**. In **b**, $^{**}p < 0.01$ by Student's t-test. Data are representative of two independents (mean ± s.d. of three mice in **b**).

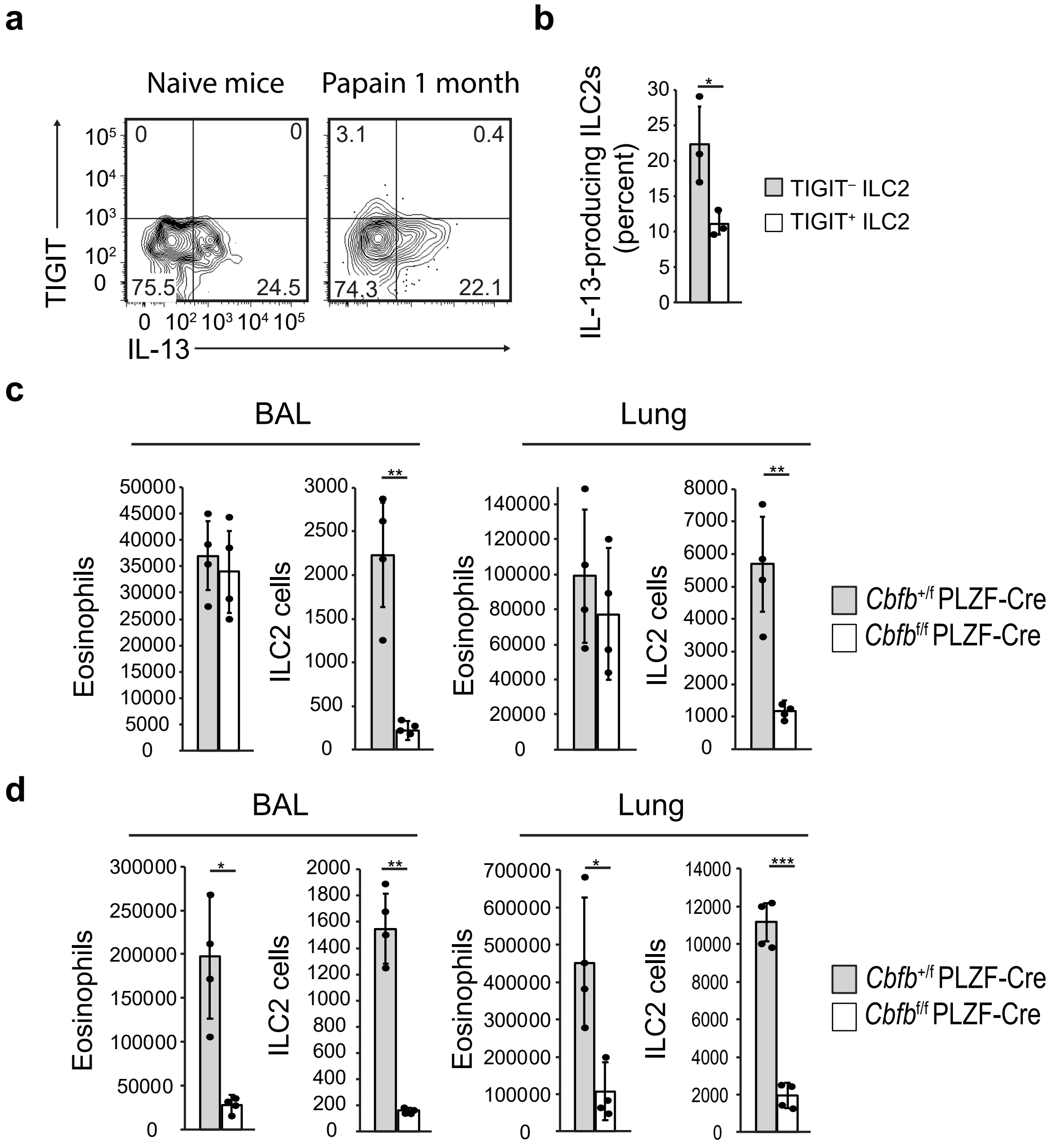

Supplementary Figure 7. Physiological roles of exhausted-like phenomenon of ILC2s in chronic allergy. **a, b** C57BL/6 mice were intranasally administered with 100 ug of papain every 3 day for a month and the lung ILC2s (CD45$^+$ CD3$^-$ CD19$^-$ NK1.1$^-$ CD11b$^-$ Gr1$^-$ Ter119$^-$ CD127$^+$ CD25$^+$ KLRG1$^+$) were stimulated with 50 ng/mL PMA and 500 ng/mL Ionomycin for 4 hours in the presence of GoldiPlug. **a** Flow cytometry analyzing production of IL-13 by the TIGIT$^+$ or TIGIT$^-$ ILC2s from the indicated mice. **b** Frequency of IL-13-producing ILC2s determined as in **a**. **c, d** Irradiated CD45.1$^+$ congenic mice were adoptively transferred with bone marrow cells of either *Cbfb*$^{+/f}$ PLZF-Cre or *Cbfb*$^{f/f}$ PLZF-Cre mice and were treated with papain every 3 days at 12-20 weeks after transfer for a month (**c**), or 3 times every 3 days in the first weak followed by 3 times every 3 days in the 4th weak after a rest of two weeks (**d**). On the day following the last papain treatment, eosinophils (CD45$^+$ CD11c$^-$ Siglec F$^+$) and ILC2s in the BAL fluid and lung were determined. Bar graphs indicate absolute numbers of eosinophils and ILC2s in the BAL and the lung from the indicated recipient mice. In **b**, **c,** and **d**, *p < 0.05, **p < 0.01 and, ***p < 0.001 by Student's t-test. Data are representative of two independent experiments (mean ± s.d. of three mice in **b** and four mice in **c, d**).

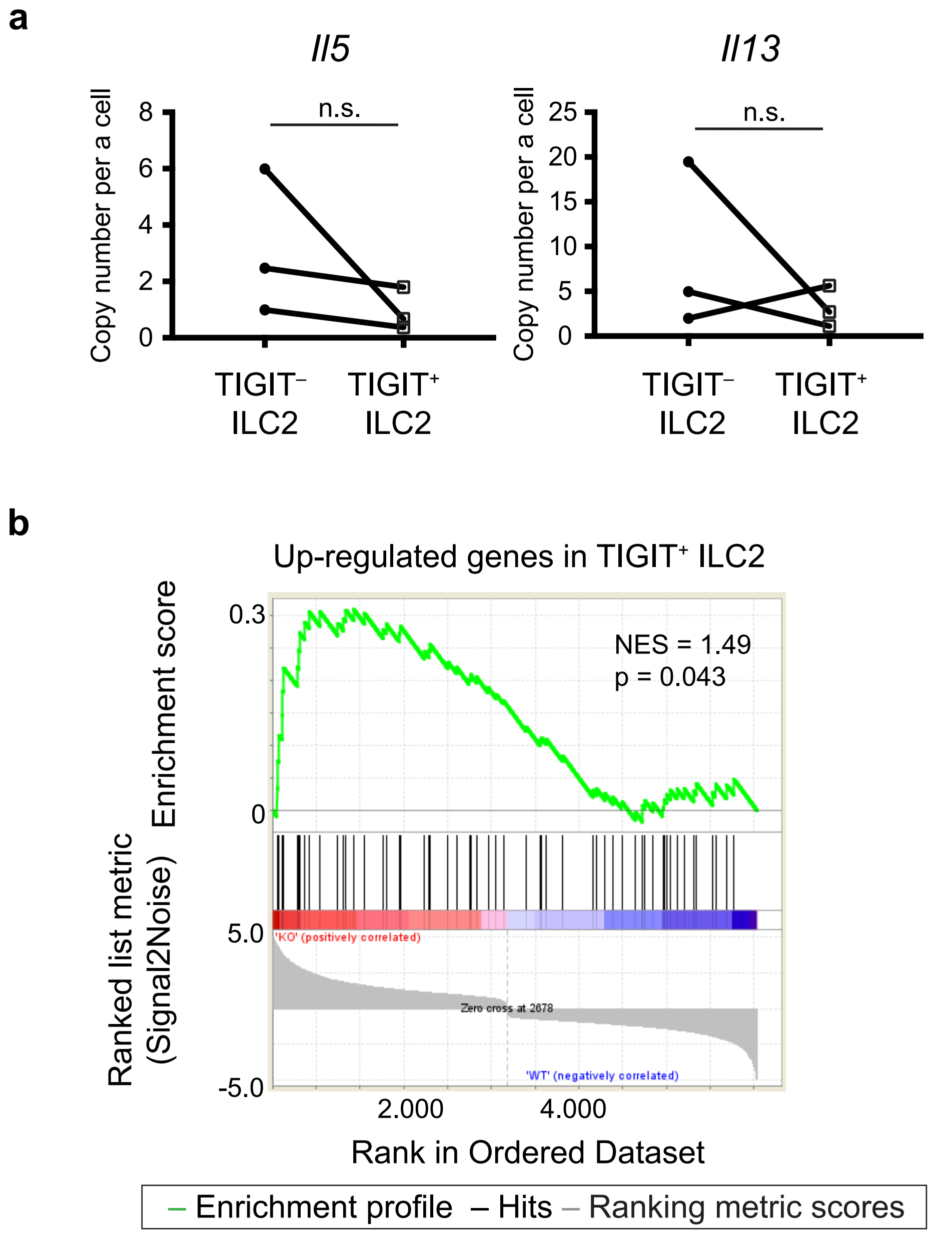

Supplementary Figure 8. TIGIT⁺ ILC2s in the BAL resemble Cbfβ-deficient ILC2s. C57BL/6 mice were intranasally inoculated with 100 ug of papain on day 0, 3, and 6. On day 7, one hundred of TIGIT⁻ and TIGIT⁺ ILC2s were sorted from BAL fluid for RNA sequence analysis with cDNA barcoding. **a** Copy number per a cell of indicated gene mRNA expression. n.s.; not significant. **b** Gene set enrichment analysis of differentially expressed genes in TIGIT⁺ ILC2s vs. TIGIT⁻ ILC2s performed on differentially expressed genes in IL-33-stimulated *Cbfβ*$^{f/f}$ PLZF-Cre ILC2s vs. *Cbfβ*$^{+/f}$ PLZF-Cre ILC2s *in vitro* as described in Fig.4i. Data are representative of two independent experiments.

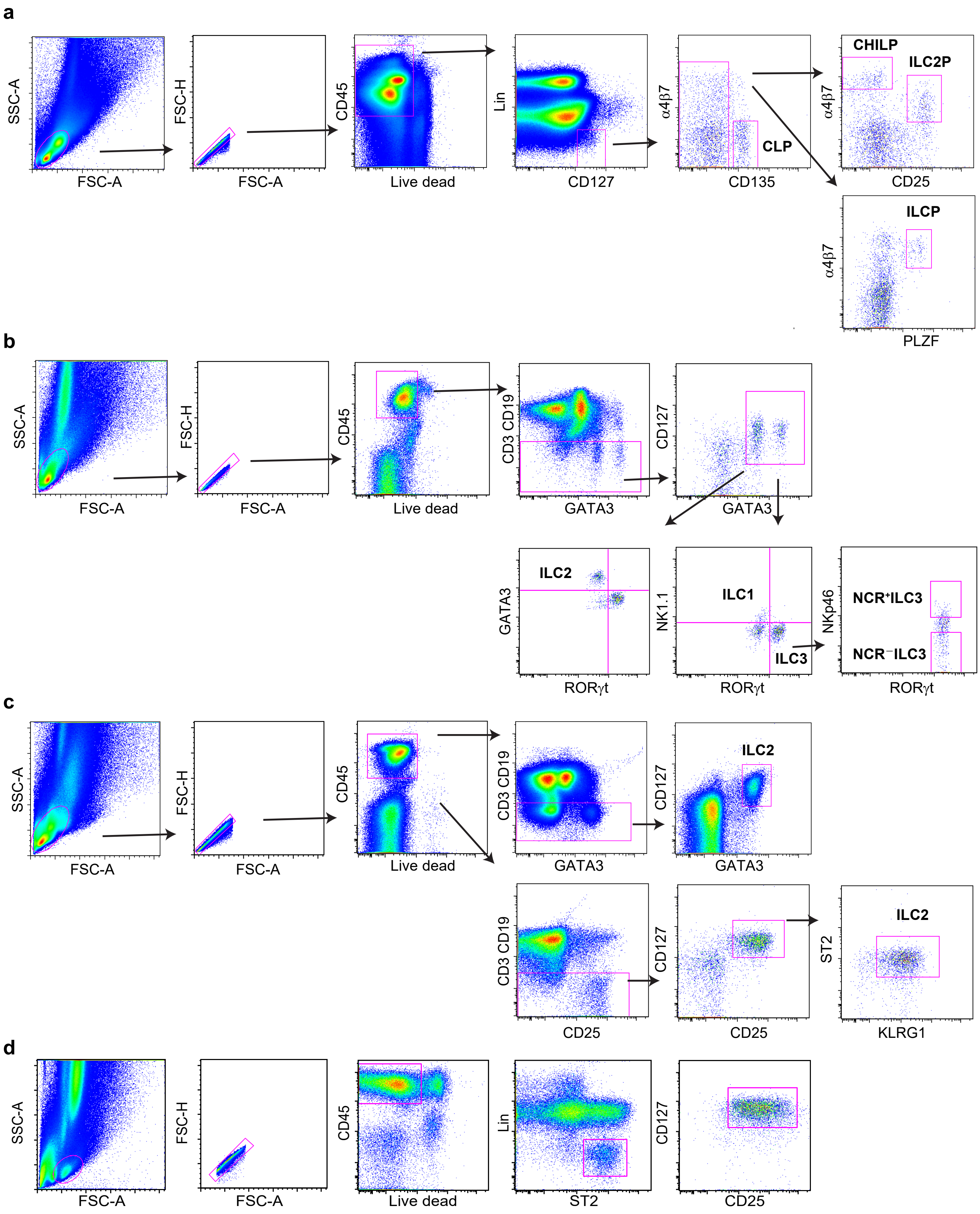

Supplementary Figure 9. Gating strategies used for cell sorting or phenotypical examination. **a** Gating strategy for CLPs, CHILPs, ILCPs, and ILC2Ps in the bone marrow presented on Fig. 1. **b** Gating strategy for ILC1s, ILC2s, NCR+ ILC3s, and NCR^{-} ILC3s in the small intestine presented on Fig. 1 and 2. **c** Gating strategy for ILC2s in the lung presented on Fig. 1, 2, 3, 4, 5, 6, 7, 8, and 9. **d** Gating strategy for ILC2s in the BAL fluid presented on Fig. 7, 8, and 9.

Supplementary Table 1. List of antibodies used in this study.

Antibody	Clone name	Company
Anti-CD3 mAb	145-2C11	eBioscience
Anti-CD19 mAb	1D3	eBioscience
Anti-CD25 mAb	PC61.5	eBioscience
Anti-KLRG1 mAb	2F1	eBioscience
Anti-CD127 mAb	A7R34	eBioscience
Anti-RORγt mAb	B2D	eBioscience
Anti-CD4 mAb	GK1.5	eBioscience
Anti-NKp46 mAb	29A1.4	eBioscience
Anti-NK1.1 mAb	PK136	eBioscience
Anti-Gr-1 mAb	RB6-8C5	eBioscience
Anti-CD11b mAb	M1/70	eBioscience
Anti-CD8a mAb	53-6.7	eBioscience
Anti-Ter-119 mAb	Ly76	eBioscience
Anti-c-kit mAb	2B8	eBioscience
Anti-sca-1 mAb	D7	eBioscience
Anti-CD135 mAb	A2F10	eBioscience
Anti-PLZF mAb	9E12	eBioscience
Anti-α4β7 mAb	DATK32	eBioscience
Anti-GATA-3 mAb	TWAJ	eBioscience
Anti-CD45.1 mAb	A20	eBioscience
Anti-CD45.2 mAb	104	eBioscience
Anti-CD49b mAb	DX5	eBioscience
Anti-CD49a mAb	Ha31/8	BD Bioscience
Anti-FceRI mAb	MAR-1	eBioscience
Anti-IgE mAb	R35-72	BD Bioscience
Anti-CD11c mAb	N418	eBioscience
Anti-IL-25R mAb	MUNC33	eBioscience
Anti-ST2 (IL33R) mAb	RMST2-2	eBioscience
Anti-Thy1.2 mAb	53-2.1	eBioscience
Anti-Thy1.1 mAb	HIS51	eBioscience
Anti-IL-5 mAb	TRFK5	eBioscience
Anti-IL-13 mAb	eBio13A	eBioscience
Anti-TIGIT mAb	GIGD7	eBioscience
Anti-IL-10 mAb	JES5-16E3	eBioscience
Anti-CD16/32 mAb	2.4g2	Bay Biosceience
Anti-Ki67 mAb	Sola15	eBioscience
Anti-GITR mAb	DTA-1	eBioscience
Anti-PD1 mAb	RMP1-30	eBioscience

Supplementary Table 2. List of primers used in this study.

Gene or transcript name	Forward primer	Reverse primer
PCR primers		
Cbfb gene (floxed region)	5’- GGTTAGGAGTCATTGTGATCA -3’	5’- CCTCCTCATTCTAACAGGAATC -3’
qPCR primer		
Cbfb gene (unfloxed region)	5’- CCATCTCCAGTCCCAAGAAT -3’	5’- AGCATTGTTGATGCTTCCAG -3’
Cbfb gene (floxed region)	5’- CGAGACCCTCACTCCAAAAT -3’	5’- GGAAAGAATCCAACCGAAGA -3’
Il10 promoter	5'- GAAGAAAATCAGCCCTCTCG -3'	5'- CGTCCGATATTTCTTGCTGA -3'
RT-PCR primers		
Cbfb with or without mutaion	5’- AACACCTAGCCGGGAATATG -3’	5’- GGTCGCCCAATGAGTTATTT -3’
qRT-PCR primers		
Cbfb (unfloxed region)	5'- CCAGACACGCTTCCAGAAC -3'	5'- CATATTCCCGGCTAGGTGTT -3'
Cbfb (floxed region)	5'- GTATGGGTTGCCTGGAGTTT -3'	5'- TCAAAGGCCTGTTGTGCTAA -3'
Il5	5’- TGGAGATTCCCATGAGCAC -3’	5’- AGGGACAGGAAGCCTCATC -3’
Il13	5’- GGATATTGCATGGCCTCTG -3’	5’- GGCGAAACAGTTGCTTTGT -3’
Il10	5’- TTGAATTCCCTGGGTGAGA -3’	5’- GGCCTTGTAGACACCTTGG -3’
tigit	5’- GTGCTGGGACTCATTTGCT -‘3	5’- GTTCTCCCAAGGCCACTTT -3’
Actb	5’- CCTGGCACCCAGCACAAT -3’	5’- GCCGATCCACACGGAGTACT -3’
