# Supplementary material for: Runx/Cbfβ complexes protect group 2 innate lymphoid cells from exhausted-like hyporesponsiveness during allergic airway inflammation
Source: Nat Commun. 2019 Jan 25;10:447. doi: 10.1038/s41467-019-08365-0 (PMC6347616; doi:10.1038/s41467-019-08365-0)
Supplement: Supplementary file 2 — Description of Additional Supplementary Files [file 41467_2019_8365_MOESM2_ESM.pdf]

Description of Additional Supplementary Files

Supplementary Data 1

List of genes regulated by GATA-3 and Cbfb in steady-state ILC2s.

Supplementary Data 2

List of genes regulated by Cbfb in ILC2s activated with IL-33.

Supplementary Data 3

List of genes differentially expressed in TIGIT+ ILC2s compared to TIGIT– ILC2s.
